# MicroRNA signatures in the pathogenesis and therapy of inflammatory bowel disease

**DOI:** 10.1007/s10238-024-01476-z

**Published:** 2024-09-11

**Authors:** Yasmin N. Ramadan, Ayat M. Kamel, Mohammed A. Medhat, Helal F. Hetta

**Affiliations:** 1 Department of Microbiology and Immunology, Faculty of Pharmacy, Assiut University, Assiut, 71515 Egypt; 2 Tropical Medicine and Gastroenterology Department, Faculty of Medicine, Assiut University, Assiut, 71515 Egypt; 3 Division of Microbiology, Immunology and Biotechnology, Department of Natural Products and Alternative Medicine, Faculty of Pharmacy, University of Tabuk, 71491 Tabuk, Saudi Arabia

**Keywords:** MicroRNA (miRNA), Inflammatory bowel disease (IBD), miRNA signature, Pathogenesis, Therapy

## Abstract

Inflammatory bowel disease (IBD) is a persistent inflammatory illness of the gastrointestinal tract (GIT) triggered by an inappropriate immune response to environmental stimuli in genetically predisposed persons. Unfortunately, IBD patients' quality of life is negatively impacted by the symptoms associated with the disease. The exact etiology of IBD pathogenesis is not fully understood, but the emerging research indicated that the microRNA (miRNA) plays an important role. miRNAs have been documented to possess a significant role in regulating pro- and anti-inflammatory pathways, in addition to their roles in several physiological processes, including cell growth, proliferation, and apoptosis. Variations in the miRNA profiles might be a helpful prognostic indicator and a valuable tool in the differential diagnosis of IBD. Most interestingly, these miRNAs have a promising therapeutic target in several pre-clinical animal studies and phase 2 clinical studies to alleviate inflammation and improve patient's quality of life. This comprehensive review discusses the current knowledge about the significant physiological role of different miRNAs in the health of the intestinal immune system and addresses the role of the most relevant differentially expressed miRNAs in IBD, identify their potential targets, and emphasize their diagnostic and therapeutic potential for future research.

## Introduction

**Fig. 1:**
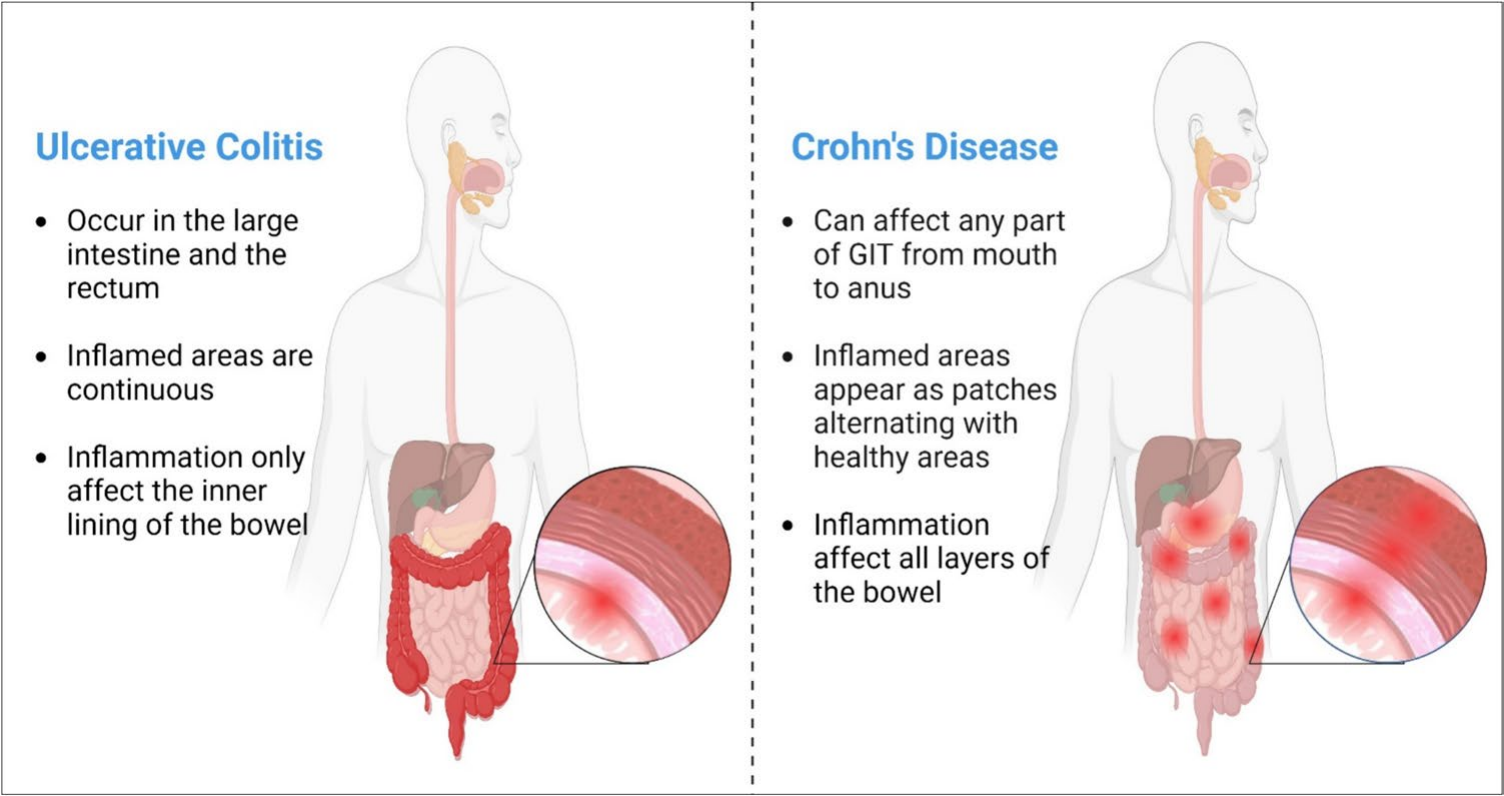
**Difference between two forms of IBD. Ulcerative colitis (left side)**; which occurs only in the colon and rectum and is characterized by the continuous appearance of inflammation and an inner layer of the bowel is involved in inflammation. **Crohn's disease (right side)**; which occurs in any part of GIT and characterized with the patchy appearance of inflammation and all layers of bowel involved in inflammation. **Created with BioRender.com**

Inflammatory bowel disease (IBD) is a chronic inflammatory and autoimmune disorder affecting the gastrointestinal tract (GIT) and is characterized by increased intestinal permeability as well as imbalanced and hyperactive immunological responses caused by environmental factors such as dietary components and gut microbiome. IBD is classified into two subtypes: ulcerative colitis (UC) and Crohn's disease (CD), each of which has unique clinical and pathological features [1]. UC is characterized by continuous and superficial inflammation of the colon or rectum mucosa that may result in erosion and ulcers (Fig. 1). On the other side, CD affects any part of the GIT, from the mouth to the anus, and is characterized by discontinuous transmural inflammation that affects all layers of the intestinal wall (Fig. 1) [2], and may be complicated with time into abscesses, strictures, or fistulas [3, 4]. CD and UC mostly affect adolescents, resulting in abdominal pain, malabsorption, bloody diarrhea, weight loss, fatigue, and decreased quality of life. Prolonged and uncontrolled inflammation also raises the chance of colorectal cancer (CRC) and increases the mortality rate to 10–15% [5].

**Fig. 2:**
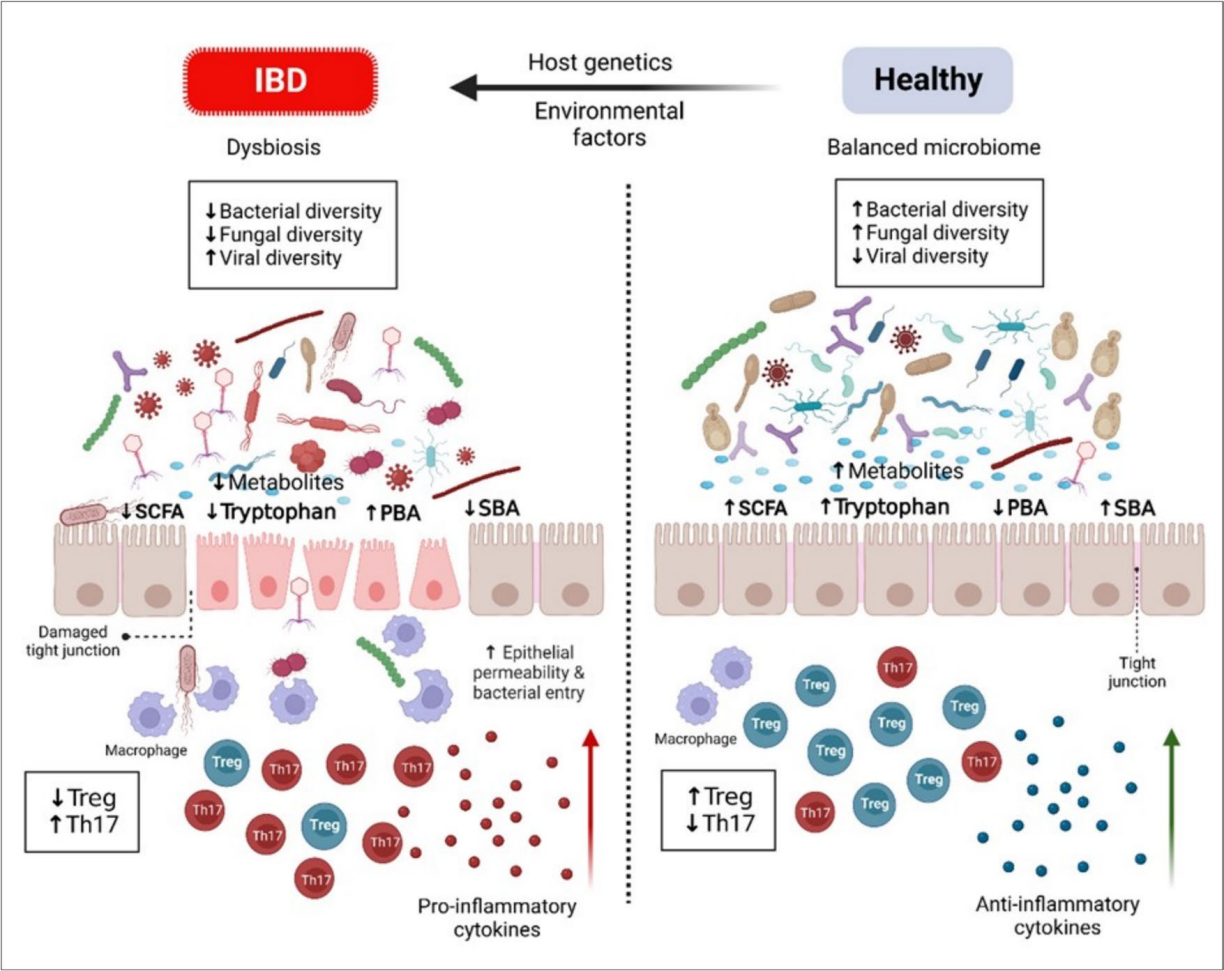
**Hypothesized pathogenesis of IBD.** It is speculated that IBD is a multifactorial disease and may be triggered by a complex interaction between immune, genetic, and environmental factors, including gut microbiome. In a healthy state, diversity, and eubiosis of the gut microbiome largely participate in the health of the intestinal barrier and tight junction, mainly through their metabolites. This keeps the balance between immune cells with the upregulation of anti-inflammatory Treg cells. In the IBD case, dysbiosis of gut microbiome is persistent and leads to damage to intestinal barrier and tight junctions with a subsequent increase of intestinal permeability for pathogens and triggering of immune cells and inflammatory reactions. **Created with BioRender.com**

Since the exact pathogenesis of IBD is unclear, it is speculated that it may be triggered by an interaction between immune, genetic, and environmental factors, including gut microbiome (Fig. 2) [2]. Around 240 genetic susceptibility loci for IBD have been discovered by genome-wide association studies (GWASs) [6–9]. Growing molecular studies revealed that microRNA (miRNA) plays a significant role in the pathogenesis of IBD [10].

miRNAs are short (~ 22 nucleotides), non-coding, single-strand RNA molecules that regulate gene expression to affect several biological processes [11]. They work by binding to the 3' untranslated region (UTR) of a target mRNA, preventing translation and limiting its expression [12]. Recent research findings indicate that miRNAs may have a positive or negative impact on the incidence and progression of IBD [13–16]. Additionally, miRNAs serve as therapeutic targets and biomarkers for diagnosis. miRNAs may help distinguish between UC and CD, in addition to being used as biomarkers of response to therapy, and disease activity, and possibly be used as predictive indicators of disease severity and the development of complications such as stenosis, penetrating disease, as well as CRC [17, 18]. In this regard, this review aims to give detailed insights about miRNA signatures in pathogenesis and differential diagnosis of IBD.

## miRNA: general overview

miRNA is a kind of short noncoding RNA that is about 18–22 nucleotides long [19, 20]. miRNAs were initially discovered in 1993 while studying *Li*n-4 gene in the nematode model, *Caenorhabditis elegans*, to find abnormalities in postembryonic maturation [21]. The ability of lin-4, the first miRNA to be identified, to downregulate the nuclear protein *lin-14* was discovered to be responsible for starting the second stage of larval development [21, 22]. In 2000, Let-7, a second miRNA discovered in *C. elegans*, seemed to be extensively conserved throughout creatures including humans [23, 24]. Based on historical data, miRNAs can significantly suppress the expression of certain genes [25, 26]. Within a particular cell type, a miRNA can target numerous mRNAs, and one mRNA is frequently the target of many miRNAs since complete complementarity is not necessary for miRNAs to recognize their targets [27]. Therefore, almost 30% of protein-coding genomes are regulated partially by miRNAs [28].

miRNAs affect several physiological processes, such as cell cycle regulation and homeostasis, cell survival, differentiation, expansion, and apoptosis. In addition, some miRNAs influence the differentiation of cells in the gut epithelium [25, 26]. As a result, miRNAs possess a significant role in the control of a variety of immune-mediated diseases, including IBD [27, 29–33].

**Fig. 3:**
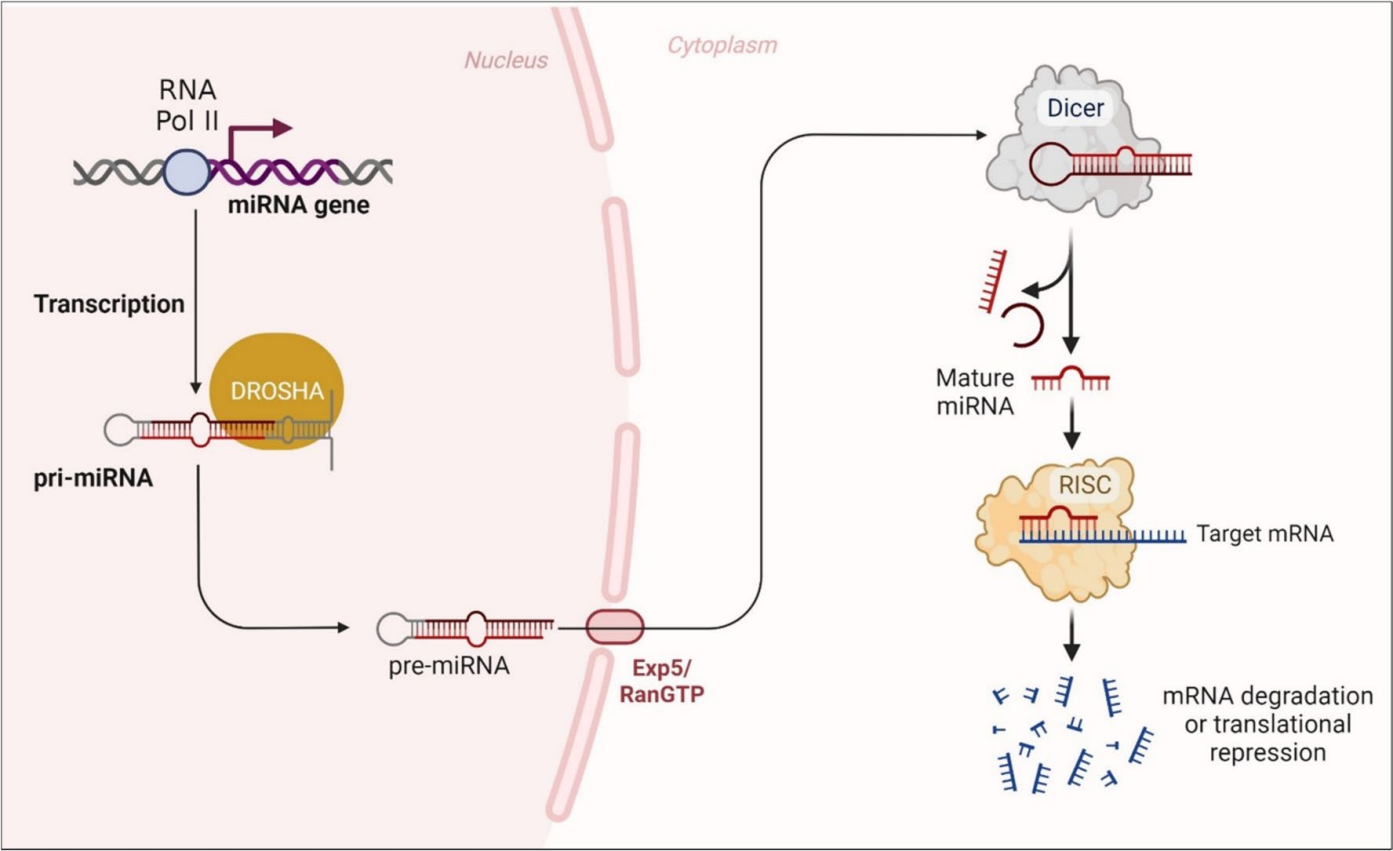
**A schematic representation of microRNA biosynthesis**. miRNA transcription is first started in the nucleus where miRNA is transcribed into primary transcript (pri-miRNA) by RNA polymerase. Then, a protein complex made up of the RNAse-III, Drosha, and DiGeorge critical region 8 (DGCR8) cleaves pri-miRNA, producing precursor miRNA (pre-miRNA), a chain of 60–70 nucleotides. After these, pre-miRNA is transported to the cytoplasm via the Exportin-5 (Exp5)—RanGTP complex. Pre-miRNA is finally cleaved into its mature state by the RNase III enzyme, dicer, which is then permanently integrated into an RNA-induced silencing complex (RISC). The RISC binds to complementary sequences in the 3′- UTR of target mRNA molecules under the guidance of the miRNA, which either causes translational suppression or mRNA destruction.** Created with BioRender.com**

miRNA genes are found within the host genome. miRNA transcription is first started in the nucleus where miRNA is transcribed into primary transcript (pri-miRNA) by RNA polymerase [34, 35]. Then, a protein complex made up of the RNAse-III, Drosha, and DiGeorge critical region 8 (DGCR8) cleaves pri-miRNA, producing precursor miRNA (pre-miRNA), a chain of 60–70 nucleotides [36–38]. After these, pre-miRNA is transported to the cytoplasm via the Exportin-5 (Exp5)—RanGTP complex [39]. Pre-miRNA is finally cleaved into its mature state by the RNase III enzyme, dicer, which is then permanently integrated into an RNA-induced silencing complex (RISC). The RISC binds to complementary sequences in the 3′-UTR of target mRNA molecules under the guidance of the miRNA, which either causes translational suppression or mRNA destruction (Fig. 3) [11, 40]. Throughout the miRNA biogenesis process, several variables may impact the stage of maturation of miRNA. These include controlling transcription, editing, controlling the turnover of miRNA, and cleaving stem-loop structures via the enzymes Drosha and Dicer. All of these processes contribute to a signaling network that adjusts gene expression as a consequence of environmental or cellular alterations [18].

Remarkably, miRNAs are found in a variety of bodily fluids, including cerebrospinal fluid, milk, saliva, feces, and urine, in addition to circulating in the human bloodstream in a stable form [41–44]. Since miRNAs play a role in the induction and progression of several illnesses, and since certain miRNAs are pathology-specific [45], research has been conducted on how variations in miRNA expression patterns may be used for prognostication, medication response prediction, and early diagnosis.

## The significance of miRNAs in intestinal immune system regulation

### Innate immune system

The innate immune system serves as the initial barrier of defense, responding quickly and non-specifically to immunological stimuli. Furthermore, the innate immune system communicates with and regulates the acquired immune system. Prior research has demonstrated the functions of miRNAs in controlling the innate immune system of the gut.

**miR-29** was demonstrated to possess a significant impact in regulating dendritic cell activity in the gut [46]. Brain et al. [46] showed that miR-29 downregulates interleukin (IL)-23 through binding IL-12p40 mRNA directly and IL-23p19 mRNA indirectly in dendritic cells, resident in the gut, in response to intracellular microbe detector, nucleotide-binding oligomerization domain containing 2 (*NOD2*). Consequently, it was proposed that miR-29 might inhibit intestinal dendritic cells' proinflammatory function. Additionally, the authors demonstrated that animal models lacking miR-29 and having high levels of IL-23 in their intestines had worsened experimental colitis.

**miR-223** was suggested to regulate the intestinal dendritic cells and macrophages. In this regard, Zhou et al. [266] found that intestinal dendritic cells and macrophages in miR-223-lacking mice exhibited a strong proinflammatory behavior. In the same study, the authors discovered that miR-223 targets the mRNA for CCAAT/enhancer binding protein β (C/EBPβ). Therefore, it was concluded that miR-223 directly targets C/EBPβ mRNA to inhibit the proinflammatory characteristics in intestinal dendritic cells and macrophages. According to Neudecker et al., mice lacking miR-223 exhibited exacerbated experimental colitis along with stimulation of the nucleotide-binding domain leucine-rich-containing family pyrin domain-containing-3 (NLRP3) inflammasome. Furthermore, colitis aggravation and NLRP3 stimulation were seen in animals lacking the miR-223 binding region in the NLRP3 3′ UTR. Amazingly, the colitis was reduced by the injection of miR-223 mimic.

**miR-146b** is thought to control the polarization of macrophages in the gut [47, 48]. According to a recent study [49], miR-146b was demonstrated to be downregulated in IBD mice and LPS-induced macrophages. Subsequent analysis revealed that miR-146b exerts its inhibitory effect through interaction with its target gene, Fibrinogen Like 2 (*FGL2*), as well as that FGL2 mediated the triggering of p38-MAPK, NLRP3, and NF-κB-p65. Consequently, it was confirmed that miR-146b could reduce M1 macrophage polarization and improve inflammatory behavior by blocking FGL2 in vitro. Furthermore, miR-146b overexpression reduced intestinal damage in vivo in IBD mice [49]. Peng et al. [50] demonstrated that IL-10 and LPS stimulated the production of miR-146b in macrophages and that IL-10-deficient macrophages showed decreased miR-146b expression. They also demonstrated that the miR-146b and mRNAs of interferon regulatory factor 5 (IRF5) may coexist on the same RISC, and miR-146b transfection mimic reduced LPS-induced IRF5 protein production and M1 macrophage activation, indicating that miR-146b targets IRF5 mRNA. Moreover, animals lacking miR-146b showed improved polarization of M1 macrophages. Based on these results, the authors hypothesized that the control of M1 macrophage activation in the gut is mostly dependent on the IL-10-miR-146b-IRF5 axis.

Reportedly, the activity of innate immune cells, such as neutrophils, natural killer cells, and innate lymphoid cells, is regulated by other miRNAs, including miR-20a, miR-34a, miR-24, miR-183, miR-150, and miR-155 [51]. More research is needed to determine if these miRNAs possess significance in the innate immune system of the gut.

### Acquired immune system

#### T-cell

miRNAs have a role in acquired immune cell (T- and B-cell) development as well. It has been demonstrated that the expression of miRNA was notably downregulated in effector T-cells that were actively dividing, while it was greatest in nonreplicating naïve T-cells and relatively inactive memory T-cells. According to recent investigations, the intestinal-acquired immune system is substantially regulated by miRNA-induced gene silencing.

Wang et al. [52] demonstrated that miR-34a suppresses Th17 cell development and proliferation in the large intestine by targeting the mRNAs of the IL-6 and IL-23 receptors, and it also inhibits Th17 migration to the epithelium by targeting the mRNA of chemokine (C–C motif) ligand 22 (CCL22). According to Takahashi et al. [53], miR-10a, which is particularly abundant in regulatory T (Treg) cells, is triggered by transforming growth factor-β (TGF-β) and retinoic acid. It targets nuclear receptor co-repressor 2 (Ncor2) mRNA and B-cell leukemia/lymphoma (Bcl) 6 mRNA in the Peyer's patches of the small intestine, attenuating the transformation of inducible Treg cells into follicular helper T (Th) cells. Additionally, they demonstrated that miR-10a inhibited Th-17 cell development, suggesting that miR-10a may have an anti-inflammatory role. In contrast, a recent study by Yang et al. [54] found that the CD4 + T cells miR-10a-deficient mice were less vulnerable to intestinal inflammation induced by dextran sulfate sodium (DSS). Additionally, they found that miR-10a reduced the production of IL-10 in the intestinal CD4 + T cells through targeting the *Prdm1* gene, which encodes transcription factor Blimp1. A study by Ge et al. [55] showed that IBD patients' colons have downregulated miR-125a, which is linked to suppression of proinflammatory cytokine secretion via targeting a transcription factor, E26 avian leukemia oncogene 1, 5′ domain (ETS-1), mRNA in CD4 + T cells. They also demonstrated that a lack of miR-125a aggravated the intestinal inflammation induced by trinitrobenzene sulphonic acid.

There is debate over the function of miR-155 in the T-cell response concerning intestinal inflammation. miR-155, which targets IL-2-inducible T-cell kinase mRNA, has been identified by Das et al. [56] to be implicated in TGF-β-induced inhibition of intestinal T-cell activation, including interferon-γ (IFN-γ) and IL-2 generation. In comparison, Chao et al. [57] found that the DDS mice model with overexpressed miR-155 in Treg cell displayed spontaneous autoimmunity and worsening of colitis. Additionally, to further inhibit the regulatory function of Treg cells, miR-155 specifically targeted the cytotoxic T-lymphocyte-associated protein 4 (CTLA-4) mRNA in Treg cells.

Sanctuary et al. [58] noted that a reduced Treg function was seen in both humans and mice with elevated miR-106a levels. This was due to the inhibition of post-transcriptional control of IL-10 release through binding of NF-κB promoter. Thus, a lack of miR-106a resulted in increased production of IL-10 and induction of Treg cells, as well as a reduction in intestinal inflammation by blocking the proliferation of Th1 and Th17 cell subsets in the intestinal lamina propria.

Mikami et al. [59] discovered that miR-221 and miR-222 are essential components for the intestinal Th17 cell response which are activated upon IL-23 stimulation to limit the extent of the proinflammatory response. The authors reported that miR-221 and miR-222 targeted the mRNAs of the MAF bZIP transcription factor (Maf) and the IL-23 receptor to inhibit the proliferation of intestinal Th17 cells upon IL-23 stimulation. Any loss of expression of miR-221 and miR-222 increased the susceptibility of mucosal barrier damage in mice models. Consequently, it was proposed that miR-221 and miR-222 affect the proinflammatory Th17 cell response in the gut by acting as negative feedback regulators downstream of IL-23.

#### B-cell

Intestinal B cells also have a role in miRNA silencing, in addition to T cells. Most miRNAs expressed in B cells are unique to different developmental stages, so, variations in miRNA expression can be utilized to categorize B-cell subpopulations [60]. Zhou et al. [61] discovered that miR-150 controls the transformation of the pro- to the pre-B-cell stage, and that upregulation of this miRNA suppresses B-cell growth. Vigorito et al. [62] revealed that miR-155 is involved in the control of humoral immunity by boosting the generation of high-affinity IgG antibodies by B2 cells through binding to its target, Pu.1 transcription factor. Moreover, Casali et al. [63] showed that miR-146a binds to the mRNA of decapentaplegic (Smad)2, Smad3, and Smad4; this reduces class-switch recombination (CSR) to immunoglobulin A (IgA). They also demonstrated that there were more IgA + B cells in the gut of mice lacking miR-146a.

## miRNAs and autophagy in IBD

**Fig. 4:**
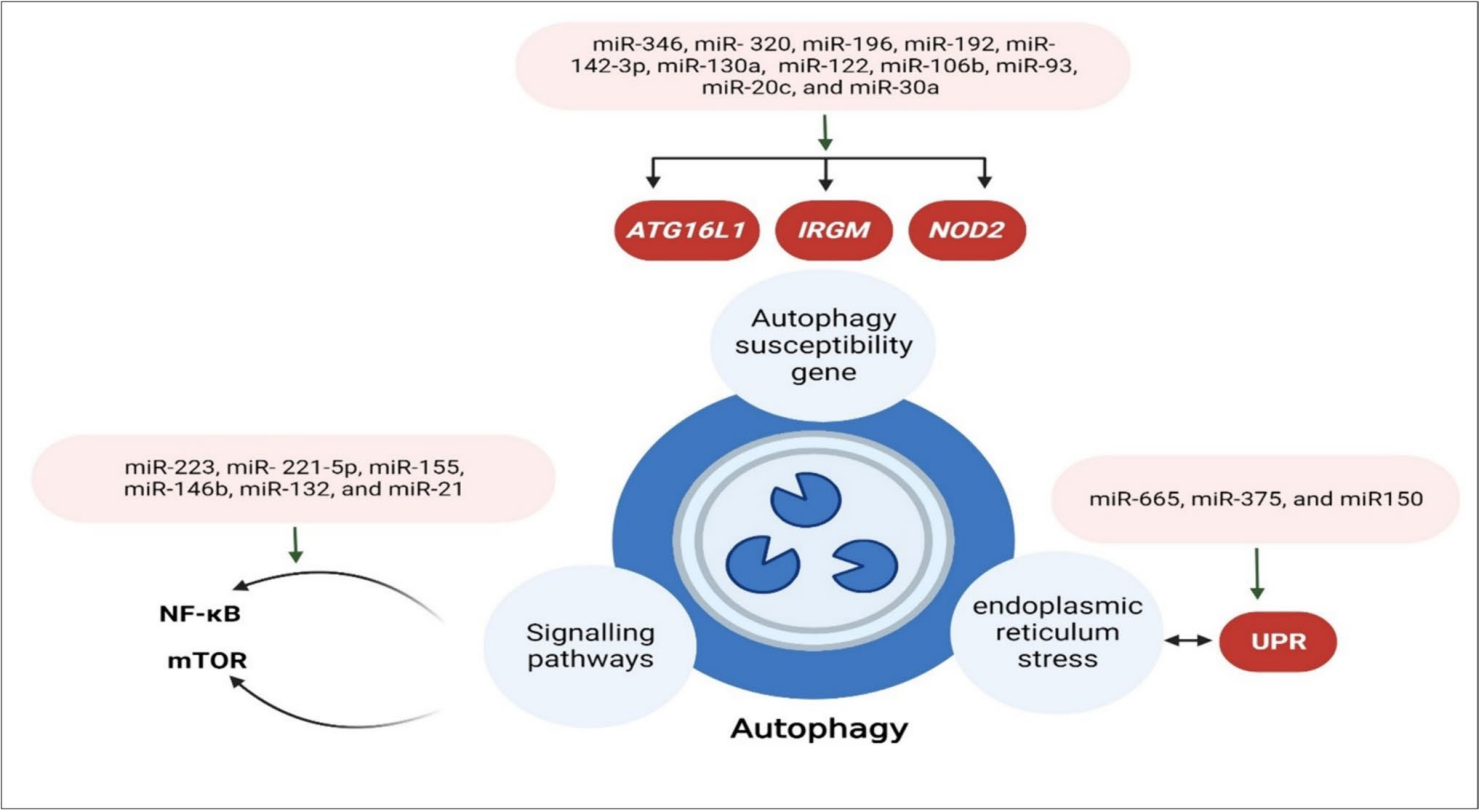
**miRNAs influence cell autophagy during IBD via several molecular pathways. a** miRNAs interact with autophagy genes associated with IBD, including *ATG16L1*, *IRGM*, and *NOD2*, to modify intestinal barrier integrity and innate intestinal immunity. **b** miRNAs regulate autophagy via modifying the unfolded protein response (UPR) during endoplasmic reticulum stress, which causes intestinal fibrosis in IBD patients. **c** miRNAs modulate NF-κB and mTOR signaling pathways that influence inflammatory mediators and intestinal autophagy. **Created with BioRender.com**

The autophagy process is well known for maintaining cellular homeostasis [64] and for being crucial to host defense, particularly in controlling inflammation [65, 66]. Any abnormalities in this process can result in several issues, such as intracellular pathogen clearance, innate immune dysfunction, as well as intestinal epithelial dysfunction [67, 68]. Recent study has also shown that miRNAs play essential roles in IBD and regulate autophagy via many cellular pathways.  miRNAs can modulate intestinal barrier integrity and innate intestinal immunity through interaction with autophagy genes involved in IBD such as *ATG16L1*, *IRGM*, and *NOD2* (Fig. 4). These miRNAs play a role in autophagy by modulating the unfolded protein response (UPR) during endoplasmic reticulum stress, thus leading to intestinal fibrosis in IBD patients [69]. Research on cellular pathways has revealed that miRNAs can influence inflammatory mediators and pro- or anti-inflammatory effects by controlling NF-κB and mTOR signaling, which in turn can stimulate or inhibit intestinal autophagy [70, 71] (Fig. 4).

## miRNAs and intestinal epithelial barrier in IBD

The intestinal epithelial barrier is a single layer of cells that prevents the body's immune cells from attacking millions of commensal microbes resident in the gut of healthy adults. It is a special structure, and the small and large intestines have very different cellular compositions that change over intestinal lengths [72]. The intestinal barrier (IB), which is made up of junctional complexes and intestinal epithelial cells (IECs), is a selectively permeable membrane that helps to sustain intestinal homeostasis. As a result, loss of IB function is linked to gastrointestinal disorders such as IBD [73]. Numerous unique IECs are found in the intestinal epithelium, including enterocytes (which aid in the absorption of nutrients), enteroendocrine cells (which secrete hormones), goblet cells (which produce mucus), Tuft cells (chemosensory cells) and Microfold (M) cells (which aid in the uptake of antigens) [74]. Additionally, the small intestine contains Paneth cells that produce proteins like epidermal growth factor (EGF) to preserve the stem cell niche and promote intestinal regeneration [75]. Moreover, Paneth cells release antimicrobial peptides such as defensins and produce genes including *IRGM1*, *NOD2*, and *ATG16L1*, which are dysregulated in CD patients. Likewise, goblet cell malfunction has been linked to problems in mucus synthesis and increased vulnerability to developing UC [72].

Several studies have reported that miRNA possesses a significant role in the proliferation, differentiation, and repair of IEC [76–82] as well as the formation and regulation of tight junctions (TJ) [83–89]. Thus, miRNA is considered one of the most important players in controlling IB integrity and permeability. To illustrate, the antibacterial capabilities of Paneth cells were boosted, and IEC proliferation and turnover were increased upon deletion of IEC-specific miR-802 in mice models. This is due to the binding of miR-802 to its target, Tmed9, which promotes defensin and Wnt secretion from Paneth cells [76].

Intestinal membrane disruption is a major contributor to the pathophysiology of IBD. It is well recognized that a key pro-inflammatory cytokine in the etiology of IBD is TNF-α. So, several in vitro studies have been carried out employing intestinal epithelial cells to cause TNF-α-induced damage [90]. It is well established that miR-191a [91] and miR-212 [92] erode IB integrity. Indeed, in vitro investigations have demonstrated that their mimics suppress the production of zonula occludens (ZO)-1, which is a key element of the tight junction connecting the IEC. Zou et al. [93] measured the interaction of miR-675 in colon cells in vitro and discovered that miR-675 destabilized the ZO-1 and E-cadherin mRNA, resulting in decreased production of vital proteins for intercellular tight junctions. Aquaporin 3 (AQP3) is an additional essential protein in IB. According to Zhi et al., [94] miR-874 increases paracellular permeability in vitro by downregulating the AQP3 protein via targeting its 3'UTR.

These findings, taken together, suggest candidate miRNAs that could be targeted to preserve IEC-mediated gut homeostasis, regulate tight junctions, and support barrier function in different GIT disorders such as IBD.

## miRNAs and gut microbiota

Interestingly, miRNA and microbiota interact reciprocally. Although exciting research on miRNAs has increased greatly in recent years, pointing to these tiny molecules as key players in the host-microbiota connection, the precise mechanisms by which miRNAs are implicated in IBD or dysbiosis remain unknown. On one hand, host miRNA may play an important role in IBD pathogenesis by modulating the gut microbiota. Conversely, the gut microbiota may influence the expression of host miRNAs, leading to intestinal epithelial disruption, impaired autophagy, and immunological hyperactivation [20, 95, 96].

In 2011, Dalmasso et al. [97] reported one of the first proof of this interaction. They investigated the effects of introducing the microbiota of pathogen-free mice into germ-free mice. Such colonization resulted in differential expression of miRNA and host genes in both the ileum and the colon. Then, Nguyen et al. [98] studied the mechanism by which some pathogenic bacteria such as adherent-invasive *Escherichia coli* (AIEC) alter the expression of miRNAs in CD patients. They found that AIEC infection increases the expression of miR 30C and miR130A, which reduces the expression of autophagy proteins (ATG5 and ATG16L1) and inhibits autophagy, resulting in an increase in intracellular AIEC and an inflammatory response. Additionally, Viennois et al. [99] reported that the gut microbiota influences fecal miRNAs such as let-7, miR-148, miR-21, and miR-196, whose levels are connected with the microbiota composition and inflammatory potential. Similarly, Tomkovich et al. [100] descried the relationship between fecal miRNAs and the abundance of particular bacterial taxon. They found that some of these miRNAs affected host genes and others affected bacterial genes, demonstrating a complex bacteria-miRNA-host connection. Furthermore, Johnston et al. [101] demonstrated that miR-21 expression boosts intestinal inflammation after altering the composition of the intestinal microbiota. They also established that the absence of miR-21 protects from colitis by decreasing Bacteroidetes and increasing protective Firmicutes and *Clostridia*.

Thus, miRNAs could serve as therapeutic agents (through mimicking or blocking approaches), acting on the host and/or the gut microbiota to manage IBD patients. Furthermore, diets enriched with prebiotics or probiotics should be considered, as they may help in altering the intestinal microbiota and regulating miRNA expression, to alleviate the intestinal inflammatory process in IBD patients. All of these approaches, however, require additional research and study in order to be properly understood and applied in the future.

## miRNAs and differential diagnosis of IBD

In 2008, the first miRNA fingerprinting study of IBD was conducted. Biopsy samples from patients with irritable bowel syndrome (IBS), microscopic colitis, infectious colitis, chronic active CD (aCD), active UC (aUC), and inactive UC (iUC) were compared with those from healthy controls [102]. Patients with aUC had differing expression levels of 11 miRNAs than the control group. miR-16-5p, miR-21-5p, miR-23a-5p, miR-24-3p, miR-29a-3p, miR-126-3p, miR-195-5p, and let-7f-5p were substantially elevated, while miR-192-5p, miR-375-3p, and miR-422b-5p were dramatically decreased. Later, several investigations were carried out to describe these changes in miRNA expression [103–105], and as a result, a number of these miRNAs were proposed as putative markers for UC and CD in colon biopsies as well as non-invasive samples including blood, feces and saliva [13, 14, 106–108]. In individuals with active colonic or ileal CD, differential miRNA expression was seen in tissues from various intestinal sites. In comparison to the colon, the terminal ileum showed upregulation in miR-22, miR-31, and miR-215 and downregulation in miR-19b, indicating that unique inflammation-related gene expression in each IBD subtype may be modulated by miRNAs [105].

It should be highlighted that several parameters vary between studies, including medication, inflammatory condition, disease duration, anatomical biopsy sites, various healthy control groups, and miRNA profiling technologies, therefore the results may be inconsistent and confusing.

### The most significantly differentially expressed miRNAs

In order to develop miRNA-based new diagnostics and therapies for IBD, it is critical to understand how miRNA expression variations correlate with disease type, the underlying processes that control miRNAs, the targeted genes, and their interaction. Regardless of the variability of miRNAs that are differentially expressed in IBD, 66 miRNAs were found by literature and meta-analysis to be significant candidates for therapeutic or diagnostic uses [109]. In the next section, we will focus on miRNAs that have been experimentally observed to particularly target IBD-related genes [110, 111].

#### Proinflammatory miRNA

#### Let-7i-5p

Let-7i-5p is a regulator of IL-6 and toll-like receptor 4 (TLR4), both of which are crucial for cytokine-mediated responses [112]. TLR4 mRNA and protein levels were downregulated in the THP-1 cell line transfected with let-7i-5p mimics [113]. In allergic inflammation, Let-7i-5p appears to help cells reset their protein composition in response to outside stimuli, however, the precise process is yet unknown [114]. Let-7i-5p functions as a major regulator of inflammation, fibrosis, hypertrophy, and cell death via controlling collagens, IL-6, IGF-1, caspase-3, and TGF-βR1 [112].

#### miR-16

miR-16 may have a role in the regulation of the NF-κB pathway UC by targeting the mRNA of the adenosine A2a receptor (A2aAR), NF-κB inhibitor. At the post-transcriptional level, miR-16 inversely controlled the expression of A2aAR. Moreover, colonic epithelial cells transfected with miR-16 mimics expressed pro-inflammatory cytokines, such as IFN-γ and IL-8, and nuclear translocation of NF-κB p65 protein. These harmful effects could be reversed by treating the cell with anti-miR-16 [115].

#### miR-19a-3p and miR-19b-3p

MiR-19a-3p and miR-19b-3p were identified as putative pathogenic indicators by serum miRNA screening of CD patients with and without strictures. Decreased levels of miR-19a-3p and miR-19b-3p were shown to be substantially associated with CD patients developing strictures, regardless of location, age, gender, illness duration, or activity [116]. Furthermore, it has been documented that miR-19a-3p increases IFN-α and IL-6 signal transduction by reducing Suppressors of cytokine signaling 3 (SOCS3) expression [117].

#### miR-21-5p

miR-21-5p was demonstrated to have a critical role in cytokine modulation [118], adaptive immune responses [119], colon epithelial cell hemostasis [120], as well as complications associated with IBD [121, 122]. Additionally, it has been shown that miR‐21-5p increases intestinal permeability as a consequence of epithelial injury. When miR-21-5p mimics were transfected in the UC mouse mode, tight junction proteins were lost, barrier permeability increased [120], and the number of CD3^+^ and CD68^+^ cells dropped [15]. The knockout of miR-21-5p in mice models also demonstrated strong resistance to colitis produced by DSS, indicating that this miRNA has pro-apoptotic properties [15]. miR-21-5p plays a role in TLR4 stimulation and monocyte differentiation and possesses a regulatory function in innate immunity. Additionally, danger signals—such as NF-kB activators in a negative feedback loop—induce miR‐21-5p in order to stop damage [123]. Furthermore, via targeting the IL-12p35 receptor, this miRNA controls the release of IL-12 from macrophages and dendritic cells [118]. miR-21-5p was also shown to have an important role in T-cell activity, with the greatest detectable expression in effector and memory T-cells and the lowest in naïve T cells [102]. However, this miRNA may be linked to permanent IBD fibrosis, and serum levels of it are elevated in patients with severe fibrosis and dysplasia [124, 125]. It is important to note that various cellular damage models have been demonstrated to be TNF-dependent, with concurrent miR-21-5p upregulation [29].

#### miR-24-3p

It has been found that miR-24-3p influences T cell development, proliferation, and immunological responses [126]. Additionally, it has been discovered that miR-24-3p silences pro-survival genes such as *PAK4* and *Bcl-2* which results in cell death [127]. PMS2L2 overexpression induces methylation of the miR-24-3p gene, which inhibits it and inhibits cell apoptosis in UC. PMS2L2 overexpression, in response to LPS, has been demonstrated to enhance *Bcl-2* expression while inhibiting cleaved-caspase-3, cleaved-caspase-9 production, and Bax expression [128]. Moreover, via targeting furin, miR-24-3p modulates the pathway of latent TGF-1 release [129].

#### miR-29a-3p

miR-29a has a role in the pathophysiology of UC by influencing intestinal epithelial cell apoptosis through *Mcl-1*. The 3'UTR of the *MCL-1* gene contains a seven-nucleotide broad binding site for miR-29a-3p [130]. It has been demonstrated that overexpression of miR-29a-3p in colonic tissue results in increased intestinal membrane permeability. On the other hand, it has been found that miR-29a-39 targets LPL in ox LDL-stimulated dendritic cells to control the expression of scavenger receptors and the release of pro-inflammatory cytokines [131].

#### miR-30c-5p

Nguyen et al. showed that adherent-invasive *Escherichia coli* (AIEC) infection, which colonizes the ileal mucosa of CD patients, upregulates the expression of miR-30c-5p in T84 cells through nuclear factor-κB activation. Up-regulation of this microRNA decreases the level of Autophagy related 5 (ATG5) protein and hence hinders autophagy, resulting in a surge in the number of intracellular AIEC and severe inflammatory response [98]. The author documented the same effect in both human patients and mouse models. On the other hand, it is thought that miR-30c-5p regulates the differentiation of Th17 cells by targeting its negative regulators, including TGFβR2, SOCS3, FOXO3, TSC1, SMAD2, as well as SMAD4 [132]. Therefore, the number of Th17 cells may rise or fall as a result of their differential regulation. Other significant targets of miR-30c-5p include STAT1, ETS1, and BCL6.

#### miR-31-5p

MiR-31-5p was shown to target FIH-1, an inhibitor of Hypoxia-inducible factor 1 (HIF-1) protein, and demonstrated a progressive increase from normal to IBD conditions [133]. Furthermore, it has been demonstrated that suppression of miR-31-5p in keratinocytes in psoriasis suppresses NF-κB-driven promoter-luciferase activity and IL-1β, CXCL1, and CXCL5 production [134]. miR-31-5p uses STK40 as its main target to attract leukocytes and control the production of these cytokines and chemokines in endothelial cells. Additionally, by targeting and inhibiting retinoic acid-inducible protein 3 (Gprc5a), miR-315p adversely controls the production of peripherally derived Treg cells [135]. In this regard, deletion of miR-31-5p boosts this Treg cell induction and minimizes the severity of EAE in animal models. IL-13 is a vital Th2 cytokine that regulates epithelial function by binding to the IL-13 receptor A1 (IL13RA1). Gwiggner et al. [136] discovered that miR-31 and miR-155 levels are elevated in inflamed UC mucosa and both target the 3' UTR of IL13RA1. In the gut epithelial cell line, transfection of miR-31 and miR-155 mimics decreased the expression of IL13RA1 mRNA and protein, inhibited IL-13-dependent phosphorylation of STAT6, and decreased the expression of SOCS1 and CCL26. These results may exacerbate the disease condition [136]. Moreover, post-ablation epithelium with higher barrier permeability had a variable expression of miR-31-5p [137]. Most recently, Qu et al. [138] proposed that miR-31 inhibition on cytokine receptors is important for controlling inflammation and may be used as a beneficial target for developing new drugs.

#### miR-106a-5p

In both CD and UC patients, serum miR-106a-5p levels are correlated with the severity of the disease [139]. It has been demonstrated that miR-106a-5p is upregulated through T-cell activation, although the majority of miRNAs are downregulated [140]. Moreover, miR-106a-5p in macrophages can control signal regulatory protein α (SIRPα) production and, thus, SIRPα-mediated inflammation [141]. Several studies demonstrated the effect of miR-106a-5p on IBD prognosis in both human and animal models [58, 142, 143]. By using the dual-luciferase reporter (DLR) assay, Li et al. [142] found that STAT3 is a target gene of miRNA-106a-5p. Therefore, miRNA-374b-5p and miRNA-106a-5p possess a role in IBD pathogenesis by regulating IL-10/STAT3 signaling pathway. Furthermore, Sanctuary et al. [58] found that miR-106a-5p knockout can ameliorate chronic ileitis in murine models and boost the suppressive function of Treg cells. It was demonstrated that a deletion in miR-106a-5p will enhance Treg induction and IL-10 production, and attenuate colitis in T cell-restricted deficiency [144]. Under physiological settings, TGFβ seems to reduce miR-106a in order to improve Treg induction. Under inflammatory circumstances, TNF-α tends to cause overexpression of miR-106a-5p via NF-B-dependent activation of the miR-106a-5p promoter, leading to transient repression of normal immune control [58].

#### miR-142-5p and miR-142-3p

miR-142-5p is the most common isoform in thymically Tregs [145]. MiR-142-5p has been demonstrated to negatively regulate PD-L1 expression by targeting its 3-UTR [146]. It has been demonstrated that overexpression of miR-142-5p causes the upregulation of proinflammatory TNF-α and IFN-γ and the downregulation of anti-inflammatory IL-10 [109]. Han et al. [147] reported that colonic mucosa of UC patients as well as HT-29 cell lines treated with TNF-α experienced downregulation of long non-coding RNA TUG1, which is considered a negative regulator of miR-142-5p. Thus, upregulation of miR-142-5p abolishes TUG1-mediated suppression of TNF-α induced IL-6, IL-8, and IL-1β. Moreover, Xiang et al. [148] demonstrated that miR-142-5p negatively regulates the protective circular RNA CCND1. Furthermore, miR-142-5p was reported to be substantially expressed in UC, and its knockdown prevented inflammatory reactions and Caco-2 cell death triggered by LPS. Additionally, miR-142-5p was demonstrated to enhance intestinal inflammation in aUC patients by upregulating the expression of the suppressor of cytokine signaling 1 (SOCS1) and secreting more IL-6 and IL-8 cytokines [149]. Duijvis et al. [150] discovered that inhibiting miR-142-5p ameliorates colitis in animal models, potentially via activating the Interleukin-10 receptor subunit alpha (IL10RA) pathway. Moreover, miR-142-5p specifically targets and inhibits genes that encode tight junction proteins (TJPs), such as TJP1, occludin, and claudin-8 [151].

MiR-142-3p is expected to target ATG16L1, one of the most frequently found genetic variants in CD patients. It has been reported that ATG16L1 in colonic epithelial cells of CD patients is negatively regulated by miR-142-3p. Thus, upregulation of miR-142-3p decreased the expression of ATG16L1, which in turn decreased the autophagic capacity of thymic-derived Tregs. According to Li et al., [152] miR-142-3p can inhibit the stimulation of the ERK1/2 signaling pathway through downregulating *RAC1* expression, which results in a Treg function deficiency. Additionally, it has been demonstrated that miR-142-3p antagomir can impact the apoptosis, and cytokine production, as well as the regulatory function of induced Treg cells via Foxp3 expression [153].

#### miR-150-5p

miR-150-5p is thought to play a role in IBD. In IBD, miR-150-5p was reported to be upregulated [154, 155]. miR-150-5p targets c-Myb, which is markedly downregulated in the colons of UC patients and the colitis model. Thus, it has been observed that overexpression of miR-150-5p increases the damage to intestinal barrier by targeting c-Myb [156]. In this regard, Rodríguez et al. [157] observed that the *Lactobacillus fermentum* probiotic can improve dysbiosis alter the level of miR-150-5p, and restore intestinal integrity and permeability. In addition, Ishihara et al. [155] found that miR-150 knockdown stopped the proliferation of damaging Th17 cells and the onset of colitis, suggesting that miRNA may considered as a potential therapeutic target for the management of IBD.

#### miR-155-5p

miR-155-5p is a well-known proinflammatory, oncogenic miRNA that is significantly expressed in activated T and B cells in addition to macrophages. miR-155-5p is essential for a functioning immune system as it regulates the activity of DCs and lymphocytes. In GIT, aberrant miR-155 expression has been reported in several disease conditions such as IBD and CRC [158]. Previous research indicated that miR-155 is upregulated in different sample types of IBD patients [159]. Expression of miR-155-5p is elevated in antigen-presenting cells (APCs), such as macrophages and DCs, in response to inflammatory mediators such as LPS, TLR ligands, and IFN-β. Additionally, it has been discovered that antigen-stimulated T and B lymphocytes trigger the expression of miR-155-5p [156]. Furthermore, one of the primary targets of miR-155-5p is SOCS1, which is a negative regulator for the triggering of LPS-induced macrophage, antigen presentation by DCs, and JAK/STAT signal pathway [160]. In addition, miR-155 antagomir protects mice from DSS-induced colitis via modulating Th17/Treg cell balance [161].

#### miR-199a-5p

miR-199a-5p was found to be significantly elevated in the blood of aUC and aCD patients compared to healthy controls [162]. miR-199a-5p was investigated to possess a pro-inflammatory effect. Overexpression of miR-199a-5p induces the phosphorylation of STAT 1 and STAT 3 proteins [163]. miR-199a-5p was shown to be implicated in endoplasmic reticulum stress (ERS) and cell death in vitro, and its overexpression promoted ERS, weight loss, apoptosis, and UC in vivo*.* Wang et al. [164] demonstrated that these effects might be avoided by inhibiting miR-199a-5p.

#### miR-223-3p

miR-223-3p was reported to be upregulated in feces and colonic biopsies of aUC and aCD [14, 165, 166]. Schönauen et al. [106] reported that the feces of active UC patients had miR-223 elevated by over 67-fold. Furthermore, Wang et al. observed that in CD patients, miR-223 had a stronger correlation with disease activity than high-sensitivity C-reactive protein (CRP) and erythrocyte sedimentation rate (ESR). miR-223-3p is overexpressed in naïve CD4 + T-lymphocytes and has been implicated in the stimulation of granulocytes. miR-223-3p enhances the development of IBD by suppressing the expression of TJP, claudin 8 [84]. Further in vivo study found that miR-223 is a key mediator in the communication between the IL-23 pathway through targeting claudin 8 and administration of its antagomir to restore claudin 8 level, improve intestinal barrier, and ameliorate DD-induced colitis [14]. Conversely, Zhang et al. [167] found that, in DSS-induced colitis mice, miR-223 agomir led to the downregulation of Bcl-2 and Bcl-xl as well as an alleviation of colonic inflammation through inhibiting IL-6/STAT3 pathway.

#### miR-424-5p

It's been demonstrated that miR-424-5p regulates monocyte/macrophage development. PU.1 is the transcription factor that controls the increase of miR-424-5p expression. MiR-424-5p, when activated, stimulates monocyte differentiation by inhibiting NFI-A [168]. Coll et al. [169] discovered that miR-424-5p possesses pro-angiogenic properties and promotes the expression of genes involved in vessel creation and the remodeling of the vascular compartment in CD stenotic and penetrating lesions, thus promoting the progression of CRC [170].

#### Anti-inflammatory miRNA

#### miR-23b-3p

miR-23b-3p suppresses inflammatory cytokine production and NF-κB activation generated by (IL-17, IL-1β, and TNF-α,) by targeting TGF-β-activated kinase 1/MAP3K7 binding protein 2 (TAB2), TAB3, and inhibiting NF-κB kinase subunit α. This effectively suppresses autoimmune inflammation. In contrast, IL-17 promotes autoimmune inflammation by inhibiting miR-23b-3p production and increasing proinflammatory cytokine production [171].

#### miR-28-5p

The functions of miR-28-5p have been demonstrated to include cell invasion, migration, proliferation, and the epithelial-to-mesenchymal transition (EMT) [172]. miR-28-5p has the ability to suppress programmed cell death protein 1 (*PD1*) genes while also regulating PD1 + Foxp3 + and TIM3 + Foxp3 + exhaustive Treg cells [173].

#### miR-30d-5p

miR-30d-5p has been demonstrated to control lactase expression and boosts the amount of *Akkermansia muciniphila *in the gut. As a result, *Akkermansia *raises Tregs to reduce symptoms of EAE. Furthermore, oral administration of miR-30d-5p mimic decreases experimental EAE [174].

#### miR-126-3p

Few studies established that miR-126-3p may contribute to the inflammatory process and IBD pathogenesis [175], whereas other studies demonstrated that miR-126-3p may possess an anti-inflammatory effect [176–178]. Active UC tissues have been demonstrated to exhibit a noticeable decrease in IκBα, the NF-κB inhibitor. Feng et al. demonstrated that miR-126-3p, by binding to the 3′-UTR of IκBα that inhibits the NF-κB signaling pathway, plays a role in the pathogenesis of UC [175]. Conversely, Zou et al. [178] demonstrated that miR-126-3p mimics can attenuate multiple organ dysfunction through upregulation of Treg and downregulation of Th17. Furthermore, it has been shown that miR-126-3p inhibits the expression of vascular cell adhesion molecule 1 (VCAM-1), which is involved in leukocyte adherence to endothelial cells, increasing the recruitment of immune cells and aggravating inflammation [176, 179]. Most recently, Jiang et al. [180] showed that miR-126-3p not only inhibits VCAM-1 expression but also IL-1β. MiR-126-3p suppression has been observed to increase PIK3R2 expression in CD8 + T cells and modify the PI3K/Akt pathway activation, which is crucial for induction and suppressive functions of Treg cells [181].

#### miR-140-5p

miR-140-5p has been demonstrated to be downregulated in various inflammatory conditions [182–186]. Numerous miR-140-5p targets control proliferation, cell cycle, and apoptosis of cells [184]. According to research, miR-140-5p downregulates TLR-4 through direct binding of its 3′-UTR and thus prevents the release of pro-inflammatory cytokines. Furthermore, miR-140-5p has been shown to suppress IL-6 and IL-8 production via modulating TLR-4 expression [182]. Yang et al. [187] observed that downregulation of miR-140-5p elevate the level of pro-inflammatory cytokines such as IL-1β, IL-6, and TNF-α in addition to upregulation of TLR-4/NF-κB signaling pathway in an in vitro model.

#### miR-141-3p

IBD and other autoimmune disorders, such as lupus and psoriasis, are characterized by abnormal expression of miR-141-3p [188, 189]. CXCL12β, a chemokine produced by epithelial cells that control colonic leukocytic trafficking, has been demonstrated as the target of miR-141-3p. Additionally, the CXCL12β is inversely correlated to miR-141-3p. Thus, it is hypothesized that miR-141-3p's targeting of CXCL12β may affect the trafficking of inflammatory cells toward inflammatory areas. Therefore, preventing immune cell trafficking and suppressing colonic CXCL12β expression may be beneficial for treating CD [190]. Additionally, miR-141-3p has been shown to inhibit STAT4, subsequently preventing inflammatory mediators [191]. Shen et al. [192] demonstrated that miR-141-3p upregulation attenuates the intensity of chronic inflammatory pain by downregulating downstream target gene high-mobility group box1 (*HMGB1*), which in turn lowers the levels of IL-1β, IL-6, and TNF-α. Chen et al. [193] also showed that elevated expression of miR-141-3p may ameliorate the necrotizing enterocolitis by targeting the motor neuron and pancreas homeobox 1 (*MNX1*) gene, which controls the expression of oxidative stress markers such as (SOD, MPO, and MDA) and inflammatory mediators such as (IL-1β, IL-6, and TNF-α). Furthermore, miR-141-3p was found to ameliorate intestinal epithelial cell damage caused by LPS via suppressing necroptosis and inflammation mediated by receptor-interacting protein kinase 1 (*RIPK1*) [194]. Most recently, Yan et al. [195] reported that, through targeting SUGT1, miR-141-3p may also prevent colonic epithelial cell pyroptosis caused by LPS. miR-141-3p may also reduce DSS-induced UC in mice. This suggests that miR-141-3p might be developed into a nucleic acid medication for the management of UC.

#### miR-146

**miR-146a-5p** has been demonstrated to control the innate immune reactions and TNF-α cascade in skin inflammation [196]. miR-146a-5p lacking mice also experience immune system problems [197]. This miRNA controls NOD2-derived gut inflammation and reduces proinflammatory cytokines produced by activated macrophages in IBD patients [198]. miR-146a expression is triggered immediately by NF-κB binding to its promoter [199]. Consequently, elevated miR-146a suppresses TLR signaling by going after TNF receptor-associated factor 6 (TRAF6) and interleukin-1 receptor-associated kinase 1 (IRAK-1). This lowers the inflammatory mediators, IL-1β, IL-6, and TNF-α, in infected macrophages [200]. Similarly, in human intestinal epithelial cells, miR-146a-5p downregulates the IRAK1/TRAF6 signaling pathway, which in turn adversely controls the IL-1β-stimulated inflammation. As a result, miR-146a-5p could be a crucial indicator for diagnosis and a therapeutic target for IBD [201]. Additionally, in the experimental colitis model, miR-146a also targets receptor-interacting serine/threonine kinase 2 (RIPK2), a NOD-like receptor signaling mediator and restricts the secretion of Th17-driving cytokines from intestinal dendritic cells (DCs) and macrophages, including IL-1β, IL-6, and IL-23 [202]. Furthermore, in response to LPS, overexpression of miR-146a-5p in monocytes led to reduced levels of TLR4 signaling network downstream genes [203].

**miR-146b-5p** has also been reported to be downregulated in IBD and LPS-induced macrophages. miR-146b directly targets and downregulates fibrinogen Like 2 (*FGL2*) gene. Therefore, by suppressing *FGL2*-activated NF-κB/MAPK signaling pathway, miR-146b alleviates intestinal inflammation in vivo and prevents M1 macrophage polarization in vitro [49]. On the other hand, miR-146b-5p may reduce intestinal inflammation via increasing NF-κB expression as a consequence of reduced levels of the *siah2* gene, which ubiquitinates TNF receptor-related factor proteins. In turn, activation of NF-κB cascade promotes intestinal epithelial function, suppresses autophagy, decreases intestinal inflammation in DDS-induced colitis, and raises the survival rate [48]. The most recent Egyptian study demonstrated that the expression of miR-146b-5p significantly increased in CD patients compared to UC, and its expression in patient's serum increased with disease activity [143].

**miR-146b-3p**, an additional member in the miR-146 family, has been demonstrated to hinder TNF-α release, inhibit proinflammatory adenosine deaminase 2 (ADA2) [204]. Moreover, elevated STAT3 activity dramatically downregulates the expression of miRNA-146b-3p [205].

#### miR-149-5p

miR-149-5p has been demonstrated to be downregulated in IBD. Wu et al. [162] found that miR-149-5p was differentially expressed in UC and CD. miR-149-5p expression was significantly decreased in aUC patients. miR-149-5p has been demonstrated to suppress TLR-induced inflammatory cytokine production by targeting MyD88. Similarly, Luo et al. [206] reported that both miR-149 isoforms (miR-149-5p and miR-149-3p) were downregulated in IBD and may linked to the disease activity. These findings thus point to their significance as disease monitoring biomarkers. Recent research using in vitro and in vivo models of colorectal cancer (CRC) and IBD revealed that the Enterotoxigenic *Bacteroides fragilis* (ETBF) bacteria, which is closely linked to these two illnesses, adversely regulates miR-149-3p which is crucial for suppressing tumor cells [207]. Furthermore, Feng et al. [208] discovered that miR-149-3p deletion alters gut microbiome and enhances the pathogenesis of DDS-induced colitis in mice.

#### miR-192-5p

miR-192-5p was downregulated in both UC [102, 209] and CD [166, 210]. According to one study, miRNAs have important roles in CD etiology and inflammatory modulation, comparable to UC. miR-192 was shown to lower inflammatory activation by suppressing NOD2 receptor function in colonocytes [210]. On the other hand, the downregulation of miR-192 might impact the progression of CD by over-activating NOD2 through muramyl dipeptide. It has been demonstrated that miR-192-5p targets and negatively regulates macrophage inflammatory Peptide 2 Alpha (*MIP2α*) (CXCL2), a CXC chemokine that is produced by epithelial cells and is crucial for both human and mouse IBD. A miR-192-5p analogue has been shown to decrease *MIP2α* expression [102]. Furthermore, miR-192-5p controls the expression of collagen and chemokines, both of which are essential for fibrosis and inflammation [25].

#### miR-195-5p

There is a correlation between the severity of IBD and miR-195-5p level. A previous study documented that miR-195-5p is overexpressed in UC patients [102]. It has been demonstrated that overexpressing miR-195-5p decreases M1 macrophage polarization. When miR-195-5p was overexpressed, TLR2 levels in M1 macrophages activated by LPS and IFN-γ decreased. Moreover, miR-195-5p dramatically reduced the levels of TNF-α, IL-6, and IL-1β in cultures of M1-stimulated macrophage supernatant. Overall, miR-195-5p appears to be involved in the polarization of macrophages through the inhibition of TLR2 inflammatory pathway regulators [211].

#### miR-200 family

miR-200 family includes miR-200a, b and c. It has been demonstrated that miR-200b was downregulated in colonic biopsies from both UC and CD. However, miR-200a and miR-200c were downregulated in colonic biopsies of CD [212]. It is believed that the pool of activated fibroblasts in IBD fibrosis is influenced by the epithelial-to-mesenchymal transition (EMT). Typically, the miR-200 family of miRNAs inhibits the production of EMTs [212]. Multiple studies, however, found that miR-200b had a greater anti-EMT effect than miR-200a and miR-200c [212–215]. An in vitro study found that miR-200b maintains intestinal epithelium integrity by suppressing EMT and enhancing IEC proliferation [213]. Another study found that *Clostridium butyricum* restored intestinal epithelium integrity by boosting miR-200c expression [216]. Furthermore, the intestinal epithelial barrier is shielded by over-expression of miR-200b, which suppresses the TNF-α-upregulated JNK/c-Jun/AP-1 signal and IL-8 production in Caco-2 cells [217]. miR-200c-3p suppresses NF-κB inflammatory pathway in response to LPS-induced TLR4 activation [218].

#### miR-375-3p

miR-375-3p is downregulated in aUC [102] and aCD [105, 108, 166] patients' intestinal mucosa and feces. However, it was reported to be upregulated in the peripheral blood of UC and CD patients compared to the control [108]. Alam et al. [219] concluded that the downregulation of miR-375 in colon tissues may be directly related to less targeted regulation of CTGF-EGFR, with consequent elevated tissue proliferation influencing cancer progress. miR-375-3p was reported to competitively suppress the expression of TLR-4. In LPS-induced caco-2 cells, knocking down miR-375 might trigger pro-inflammatory cytokines production such as IL-1β, IL-6, IL-8, and TNF-α, and deterioration of intestinal integrity [16]. Furthermore, Cheng et al. [220] observed that miR-375-3p reduces the intensity of inflammation by targeting YAP1/LEKTI pathway.

#### miR-378a-3p

miR-378a-3p is downregulated in aUC [221] and feces of CD [166]. miR-378a-3p has been demonstrated to target and negatively regulate IL-33 [221]. Li et al. [222] showed that mesenchymal stem cells-derived extracellular vesicles (MSCs-EVs) carrying miR-378a-3p can inhibit the GATA2/AQP4/PPAR-α pathway, therefore decreasing LPS-induced apoptosis in M064 cells and preventing the development of IBD. The miR-378a-3p is found in intron 1 of the *PPARGC1B* gene, which is regulated differently in the intestinal mucosa of UC patients [223]. *PPARGC1B* protein is abundantly expressed in the intestinal epithelium and has a role in energy generation and biogenesis [224], regulation of mitogenesis, and mitochondrial metabolism [225]. Consequently, it can be said that in inflammatory mucosa, the reduction in miR-378a-3p may indicate a metabolic change, perhaps connected to an increase in energy consumption and the overproduction of ROS [226].

#### miR-532-3p

miR-532-3p was downregulated in the peripheral blood of aUC [102] and aCD [162]. miR-532-3p suppresses inflammation by inhibiting the ASK1/p38 MAPK signaling cascade in LPS/TNF-stimulated macrophages. For this reason, it has been proposed as a possible target for the treatment of inflammatory autoimmune disorders like IBD [227].

In summary, numerous miRNAs have been found since their discovery. There is growing evidence that certain miRNA expression profiles have a role in the onset and progression of IBD. The majority of studies find correlations rather than causal links between IBD and differentially expressed miRNAs. The exact role of the majority of miRNAs in IBD remains unclear since, as was previously said, very little research focuses on the underlying biological processes of the illness. Additionally, a lack of standardized study designs and varied methodologies have contributed to the lack of consistency between investigations.

We concluded that there are common miRNAs (such as miR-16, 21, 31, 155, and 223) as well as some differentially expressed miRNAs in colon biopsies, peripheral blood, feces, and saliva after comparing miRNAs in various tissues among UC or CD patients and control. These differentially expressed miRNAs may aid in the clinical diagnosis and differentiation of UC and CD. Furthermore, it is unlikely that the miRNA expression seen in colon biopsies will match the miRNA expression in peripheral blood, as that present in blood might represent expression in circulating white blood cells (WBCs) [25]. The varying evolutionary stages of IBD may also contribute to variations in the expression level of miRNA. (Table 1) and (Table 2) clarifies the differentially expressed miRNA across various sample types in UC and CD, respectively.

**Table 1:** Differentially expressed miRNA across various sample types in UC [159, 257]

Sample type	Upregulated miRNA	Downregulated miRNA	References
Peripheral blood	miR-16, miR19a, miR-21, miR-28-5p, miR-30e, miR-101, miR-103-2, miR-106, miR-142-5p, miR-146-5p, miR-151-5p, miR-155, miR-199a-5p, miR-215, miR-223, miR-340, miR-362-3p, miR-374b, miR-375, miR-494, miR-532-3p, miR-598, miR-638, miR-642, and miRplus-E1271	miR-21, miR-31, miR-146a, and miR-505	[108, 139, 162, 258–260]
Feces	miR-16-5p, miR-21-5p, miR-126, miR-155, miR-203, miR-223, and miR-1246	miR-192 and miR-320	[106, 121, 165, 209]
Saliva	miR-21, miR-31, and miR-142-3p	miR-142-5p	[108]
Intestinal biopsy	miR-15, miR-16-5p, miR-19a, miR-21-5p (-3p), miR-23a-5p, miR-24-3p, miR-29a-3p, miR-31, miR-101, miR-125b-1-3p, miR-126-3p, miR-146a-3p, miR-155-5p miR-195-5p, miR-206, miR-594 and let-7f-5p	miR-192-5p, miR-375-3p, miR-200b, miR-214-3p and miR-422b-5p	[14, 102, 108, 212, 261–263]

**Table 2:** Differentially expressed miRNA across various sample types in CD [159, 257]

Sample type	Upregulated miRNA	Downregulated miRNA	References
Peripheral blood	miR-16, miR-23a, miR-29a, miR-106a, miR-107, miR-126, miR-101, miR-146-5p, miR-146b-5p, miR-191, miR-199a-5p, miR-200c, miR-340, miR-362-3p, miR-375, miR-532-3p, miR-598, miR-642, and miRplus-E1271	miR-21, miR-31, miR-146a, miR-149, and miRplus-F1065	[106, 108, 162, 258–260, 264]
Feces	miR-15a-5p, miR-16-5p, miR-24-3p, miR-27a-3p, miR-126-3p, miR-128-3p, miR-142-5p, miR-155, miR-223-3p, miR-223-5p, and miR-3074-5p	miR-10a-5p, miR-10b-5p, miR-141-3p, miR-192-5p, miR-200a-3p, miR-375, miR-378-3p, and let-7g-5p	[106, 121, 166]
Saliva	miR-21, miR-31, and miR-142-3p	miR-26a, miR-101	[108]
Intestinal biopsy	miR-16, miR-19a, miR-21-3p, miR-23b, miR-30c, miR-31-3p, miR-101, miR-106a, miR-130a, miR-146a-3p, miR-155-5p, miR-191, miR-195, miR-223, and miR-594	miR-19b, miR-141, miR-200a, miR-200b, miR-200c, miR-375, miR-429, and miR-629	[98, 105, 108, 212, 261, 264, 265]

## miRNAs as a target for the treatment of IBD

### Preclinical animal studies

In the future, miRNAs could serve as the real therapeutic target in addition to their function as diagnostic markers and indicators of inflammatory activity [228]. Several miRNAs have been found to work on similar inflammatory pathways as some biological drugs that are approved for the management of IBD. miR-29 has been found to be a member of the miRNA family capable of downregulating pro-inflammatory IL-23, similar to Ustekinumab, a monoclonal antibody that suppresses IL12/23. Therefore, mimicking miR-29 is advised in moderate to severe CD cases. [46, 176]. miR-126 prevents leukocyte adherence to endothelial cells via regulating VCAM-1, which is a comparable mechanism of action with vedolizumab, which is additionally approved for the management of IBD [176, 229]. The miR-155 antagomir targets and negatively regulates a JAK signaling pathway regulatory protein [230], simulating the usage of JAK inhibitors presently available for the management of UC [231].

miRNAs have a specific way of action, which suggests that either blocking or increasing miRNA expression may be preferable in order to alleviate IBD. Lima et al. have provided a detailed introduction to gain-of-function strategies like using chemically generated miRNA mimics or agomirs, and loss-of-function strategies like using miRNA sponge technology or using miRNA antagomir [232].

Up till now, these two approaches have shown promising results in preclinical animal models of IBD as well as in vitro cell lines, however, clinical data is lacking. For example, the enema administration of leptosome-miR31 mimics packed into oxidized konjac glucomannan, in the DSS-induced miR-31 knockout mice model, results in an inhibition of inflammatory reaction, increases body weight and colon length, as well as promotes epithelial cell proliferation in contrast to controls [79]. While miR-31 is elevated in the clinic, it is still unknown if this slows down or speeds up the development of IBD [79]. Moreover, numerous miRNA inhibitors have been reported to upregulate the expression of TJ protein in the UC or CD animal models, including antisense miR-122a, miR-7a-5p antagomir, miR-155 antagomir, and miR-223 antagomir [14, 233–235]. According to Fang et al., miR-31-3p agomir reduced the severity of colitis produced by DSS in mice via downregulating RhoA [236].

Additionally, apoptotic genes also appear to be attractive candidates for miRNA modulation in the management of IBD. *Bcl-2* and *Bcl-XL* are known to have an anti-apoptotic effect. In this context, Chen et al. [237] reported that intraperitoneal injection of miR-16 antagomir increased the expression of *Bcl-2* and improved intestinal function in the DSS mice model. On the other hand, Zhang et al. [167] found that in the DSS mice model, miR-223 agomir led to the downregulation of *Bcl-2* and *Bcl-xl* with subsequent remission of colonic inflammation. Remarkably, miR-223 has been identified as a pro-inflammatory miRNA in several investigations [14, 238]. Therefore, further research is needed to resolve this confusion. Furthermore, additional strategies to enhance the effectiveness of miRNA in vivo administration are being investigated by researchers. According to Suri et al., [107] there are five primary methods for delivering miRNA: viral vectors, exosomes, and conjugates in addition to lipid carriers (like lipid nanoparticles and liposomes) and polymeric carriers (like cationic carriers). Ye et al. [233] discovered that administering lipofectamine-coated antisense miR-122a to colitis mice might reduce the inflammatory response. Tian et al. [79] discovered that peptosome-miR31 surrogates coated with oxidized konjac glucomannan demonstrated greater stability in comparison to polysaccharide- and liposome-based nanoparticles (NPs). Deng et al. [239] showed that intestinal macrophages are specifically targeted for mucosal regeneration in UC and colitis-associated cancer in DDS mice model by loading miR-146b mimic on mannose-modified trimethyl chitosan [MTC]-conjugated NPs. Exosomes have received the most attention in recent debate among these miRNAs. Exosomes are now commonly recognized as a natural NP carrier for targeted drug delivery, and miRNA mimics or inhibitors loaded into exosomes might be an efficient therapy for IBD [240, 241]. Cao et al. [207] discovered that enterotoxigenic *Bacteroides fragilis* might lower the amount of exosome-packed miR-149-3p in IBD patients' plasma. Notably, drug delivery via exosomes is still challenging due to several factors, including inadequate clinical-grade manufacturing, unstandardized separation and purification techniques, and low drug-loading efficiency [242]. The complex ecosystem in the GI tract of IBD patients may also restrict the efficacy of exosome-packaged miRNA mimics or inhibitors as treatments [159]. For these obstacles, we will need to produce cell-derived artificial exosomes or novel biomaterials in the near future to encapsulate miRNA mimics or inhibitors.

When comparing loss-of-function with gain-of-function approaches, replacing defective miRNA appears to be more difficult than producing anti-miRNA. Firstly, synthetic miRNA mimics must be integrated into the RISC complex to restore its biological function. Furthermore, the carrier must selectively target interested cells with sufficient quantity and efficacy to perform its action before clearance. Lastly, the most often utilized carrier is a viral vector, which is administered intravenously or colonically, through enema, and is strongly linked to toxicity and immunogenicity [243].

### Clinical trials evaluating miRNA-associated drugs

#### ABX464 for UC

ABX464, also known as obefazimod, is a small molecule that specifically increases the expression of miR-124 in immune cells. ABX464 is an oral medication that was originally developed as an inhibitor of HIV replication and enhances the production of miR-124 from the miR-124.1 chromosomal region [244, 245]. ABX464 has been demonstrated to interact with the cap-binding complex, resulting in increased expression of miR-124. Preclinical research revealed that ABX464 offered long-term protection, alleviated DSS-induced colitis in mice, and triggered the expression of IL-22, a cytokine implicated in colitis tissue healing. [246]. ABX464 was evaluated in phase 2a and 2b trials and was demonstrated to significantly ameliorate moderate-to-severe aUC when compared to placebo [247, 248]. A long-term maintenance phase was available to UC patients who finished the induction phase. For more details about the registration of the induction phase trial [249], long-term phase trial [250], and phase 2b clinical trial [251].

#### ABX464 for CD

Additionally, Abivax Company has started a phase 2a clinical trial to evaluate the efficacy and safety of ABX464 in moderate-to-severe aCD patients who have not responded well to previous treatments with amino-salicylic acid, corticosteroids, immunosuppressants, and/or biologics or who have become intolerant to them [252].

In future research, additional clinical trials should be extrapolated for IBD management, especially after demonstrating a promising effect against other disease conditions. For example; MRX34, a liposomal miR-34a mimic, in patients with advanced solid tumors [253], and Miravirsen, miR-122 antagomir, in patients with brain tumors [254] and hepatitis C virus (HCV) infection [255, 256].

## Conclusion and future perspectives

IBD is a complex and multifactorial disease. The exact etiology of IBD is still out of reach, but it is associated with genetic and environmental factors. One of the main theories for the development of IBD is the dysbiosis of the intestinal ecosystem and the disruption of the intestinal barrier. Many miRNAs are involved in the complex pathways that regulate gut microbiome, intestinal integrity, and intestinal inflammation. Moreover, it appears that there are an infinite number of molecular interactions; currently, new research indicates that some miRNAs may be targeted as therapeutic targets or diagnostic biomarkers. Mimicking or inhibiting miRNA activity may represent a promising addition to the IBD therapeutic arsenal, as they have been demonstrated to be implicated in numerous IBD pathogenic pathways. On the other hand, human studies are scarce, and they generally focus on miRNA expression in certain cells and circumstances, leaving little information regarding their dynamic changes during inflammation and in response to treatments. Future in vivo research is required to confirm results from in vitro studies and investigate the efficacy of modifying miRNA expression in IBD.

## Data Availability

No datasets were generated or analysed during the current study.

## References

[1] Oligschlaeger Y, et al. Inflammatory bowel disease: a stressed gut/feeling. Cells. 2019;8(7):659. 31262067 10.3390/cells8070659 PMC6678997

[2] Guan Q. A comprehensive review and update on the pathogenesis of inflammatory bowel disease. J Immunol Res. 2019;2019:7247238. 31886308 10.1155/2019/7247238 PMC6914932

[3] Torres J, et al. Crohn’s disease. Lancet. 2017;389(10080):1741–55. 27914655 10.1016/S0140-6736(16)31711-1

[4] Torres J, et al. ECCO guidelines on therapeutics in Crohn’s disease: medical treatment. J Crohns Colitis. 2020;14(1):4–22. 31711158 10.1093/ecco-jcc/jjz180

[5] Stidham RW, Higgins PDR. Colorectal cancer in inflammatory bowel disease. Clin Colon Rectal Surg. 2018;31(3):168–78. 29720903 10.1055/s-0037-1602237 PMC5929884

[6] Uniken Venema WT, et al. The genetic background of inflammatory bowel disease: from correlation to causality. J Pathol. 2017;241(2):146–58. 27785786 10.1002/path.4817

[7] de Lange KM, et al. Genome-wide association study implicates immune activation of multiple integrin genes in inflammatory bowel disease. Nat Genet. 2017;49(2):256–61. 28067908 10.1038/ng.3760 PMC5289481

[8] Huang H, et al. Fine-mapping inflammatory bowel disease loci to single-variant resolution. Nature. 2017;547(7662):173–8. 28658209 10.1038/nature22969 PMC5511510

[9] Ellinghaus D, et al. Analysis of five chronic inflammatory diseases identifies 27 new associations and highlights disease-specific patterns at shared loci. Nat Genet. 2016;48(5):510–8. 26974007 10.1038/ng.3528 PMC4848113

[10] Soroosh A, et al. Functional role and therapeutic targeting of microRNAs in inflammatory bowel disease. Am J Physiol Gastrointest Liver Physiol. 2018;314(2):G256–62. 29146677 10.1152/ajpgi.00268.2017 PMC5866423

[11] Bartel DP. MicroRNAs: genomics, biogenesis, mechanism, and function. Cell. 2004;116(2):281–97. 14744438 10.1016/s0092-8674(04)00045-5

[12] O’Brien J, et al. Overview of MicroRNA biogenesis, mechanisms of actions, and circulation. Front Endocrinol (Lausanne). 2018;9:402. 30123182 10.3389/fendo.2018.00402 PMC6085463

[13] Wang H, et al. Circulating MicroRNA223 is a new biomarker for inflammatory bowel disease. Medicine (Baltimore). 2016;95(5): e2703. 26844512 10.1097/MD.0000000000002703 PMC4748929

[14] Wang H, et al. Pro-inflammatory miR-223 mediates the cross-talk between the IL23 pathway and the intestinal barrier in inflammatory bowel disease. Genome Biol. 2016;17:58. 27029486 10.1186/s13059-016-0901-8 PMC4815271

[15] Shi C, et al. MicroRNA-21 knockout improve the survival rate in DSS induced fatal colitis through protecting against inflammation and tissue injury. PLoS ONE. 2013;8(6): e66814. 23826144 10.1371/journal.pone.0066814 PMC3691313

[16] Wu CP, et al. Hsa-miR-375 promotes the progression of inflammatory bowel disease by upregulating TLR4. Eur Rev Med Pharmacol Sci. 2019;23(17):7543–9. 31539144 10.26355/eurrev_201909_18871

[17] Feng Y, et al. MicroRNAs, intestinal inflammatory and tumor. Bioorg Med Chem Lett. 2019;29(16):2051–8. 31213403 10.1016/j.bmcl.2019.06.013

[18] James JP, et al. MicroRNA biomarkers in IBD-differential diagnosis and prediction of colitis-associated cancer. Int J Mol Sci. 2020;21(21):7893. 33114313 10.3390/ijms21217893 PMC7660644

[19] Ambros V. microRNAs: tiny regulators with great potential. Cell. 2001;107(7):823–6. 11779458 10.1016/s0092-8674(01)00616-x

[20] Oliveira ECS, et al. Intestinal microbiota and miRNA in IBD: a narrative review about discoveries and perspectives for the future. Int J Mol Sci. 2023;24(8):7176. 37108339 10.3390/ijms24087176 PMC10138604

[21] Lee RC, Feinbaum RL, Ambros V. The C. elegans heterochronic gene lin-4 encodes small RNAs with antisense complementarity to lin-14. Cell. 1993;75(5):843–54. 8252621 10.1016/0092-8674(93)90529-y

[22] Ruvkun G, Giusto J. The Caenorhabditis elegans heterochronic gene lin-14 encodes a nuclear protein that forms a temporal developmental switch. Nature. 1989;338(6213):313–9. 2922060 10.1038/338313a0

[23] Reinhart BJ, et al. The 21-nucleotide let-7 RNA regulates developmental timing in Caenorhabditis elegans. Nature. 2000;403(6772):901–6. 10706289 10.1038/35002607

[24] Pasquinelli AE, et al. Conservation of the sequence and temporal expression of let-7 heterochronic regulatory RNA. Nature. 2000;408(6808):86–9. 11081512 10.1038/35040556

[25] Archanioti P, et al. Micro-RNAs as regulators and possible diagnostic bio-markers in inflammatory bowel disease. J Crohns Colitis. 2011;5(6):520–4. 22115369 10.1016/j.crohns.2011.05.007

[26] Coskun M, et al. MicroRNAs in inflammatory bowel disease-pathogenesis, diagnostics and therapeutics. World J Gastroenterol: WJG. 2012;18(34):4629. 23002331 10.3748/wjg.v18.i34.4629 PMC3442200

[27] Chapman CG, Pekow J. The emerging role of miRNAs in inflammatory bowel disease: a review. Therap Adv Gastroenterol. 2015;8(1):4–22. 25553076 10.1177/1756283X14547360 PMC4265084

[28] Dalal SR, Kwon JH. The role of MicroRNA in inflammatory bowel disease. Gastroenterol Hepatol (N Y). 2010;6(11):714–22. 21437020 PMC3033542

[29] Zarjou A, et al. Identification of a microRNA signature in renal fibrosis: role of miR-21. Am J Physiol Renal Physiol. 2011;301(4):F793–801. 21775484 10.1152/ajprenal.00273.2011 PMC3191802

[30] Møller T, et al. Co-detection of miR-21 and TNF-α mRNA in budding cancer cells in colorectal cancer. Int J Mol Sci. 2019;20(8):1907. 30999696 10.3390/ijms20081907 PMC6515373

[31] Kjaer-Frifeldt S, et al. The prognostic importance of miR-21 in stage II colon cancer: a population-based study. Br J Cancer. 2012;107(7):1169–74. 23011541 10.1038/bjc.2012.365 PMC3461159

[32] Schaefer JS. MicroRNAs: How many in inflammatory bowel disease? Curr Opin Gastroenterol. 2016;32(4):258–66. 27138057 10.1097/MOG.0000000000000284 PMC5659191

[33] Mirzaei R, et al. The pathogenic, therapeutic and diagnostic role of exosomal microRNA in the autoimmune diseases. J Neuroimmunol. 2021;358: 577640. 34224949 10.1016/j.jneuroim.2021.577640

[34] Lee Y, et al. MicroRNA genes are transcribed by RNA polymerase II. Embo j. 2004;23(20):4051–60. 15372072 10.1038/sj.emboj.7600385 PMC524334

[35] Borchert GM, Lanier W, Davidson BL. RNA polymerase III transcribes human microRNAs. Nat Struct Mol Biol. 2006;13(12):1097–101. 17099701 10.1038/nsmb1167

[36] Heo I, et al. Mono-uridylation of pre-microRNA as a key step in the biogenesis of group II let-7 microRNAs. Cell. 2012;151(3):521–32. 23063654 10.1016/j.cell.2012.09.022

[37] Han J, et al. Molecular basis for the recognition of primary microRNAs by the Drosha-DGCR8 complex. Cell. 2006;125(5):887–901. 16751099 10.1016/j.cell.2006.03.043

[38] Han J, et al. The Drosha-DGCR8 complex in primary microRNA processing. Genes Dev. 2004;18(24):3016–27. 15574589 10.1101/gad.1262504 PMC535913

[39] Zeng Y, Cullen BR. Structural requirements for pre-microRNA binding and nuclear export by Exportin 5. Nucleic Acids Res. 2004;32(16):4776–85. 15356295 10.1093/nar/gkh824 PMC519115

[40] Gregory RI, et al. Human RISC couples microRNA biogenesis and posttranscriptional gene silencing. Cell. 2005;123(4):631–40. 16271387 10.1016/j.cell.2005.10.022

[41] Weber JA, et al. The microRNA spectrum in 12 body fluids. Clin Chem. 2010;56(11):1733–41. 20847327 10.1373/clinchem.2010.147405 PMC4846276

[42] Correia CN, et al. Circulating microRNAs as potential biomarkers of infectious disease. Front Immunol. 2017;8:118. 28261201 10.3389/fimmu.2017.00118 PMC5311051

[43] Galimberti D, et al. Circulating miRNAs as potential biomarkers in Alzheimer’s disease. J Alzheimers Dis. 2014;42(4):1261–7. 25024331 10.3233/JAD-140756

[44] Alamdari-Palangi V, et al. microRNA in inflammatory bowel disease at a glance. Eur J Gastroenterol Hepatol. 2021;32(2):140–8. 32558695 10.1097/MEG.0000000000001815

[45] Landgraf P, et al. A mammalian microRNA expression atlas based on small RNA library sequencing. Cell. 2007;129(7):1401–14. 17604727 10.1016/j.cell.2007.04.040 PMC2681231

[46] Brain O, et al. The intracellular sensor NOD2 induces microRNA-29 expression in human dendritic cells to limit IL-23 release. Immunity. 2013;39(3):521–36. 24054330 10.1016/j.immuni.2013.08.035

[47] Sonoyama K, Ohsaka F. Role of microRNAs in the crosstalk between the gut microbiota and intestinal immune system. Biosci Microbiota Food Health. 2023;42(4):222–8. 37791343 10.12938/bmfh.2023-027 PMC10542430

[48] Nata T, et al. MicroRNA-146b improves intestinal injury in mouse colitis by activating nuclear factor-κB and improving epithelial barrier function. J Gene Med. 2013;15(6–7):249–60. 23813877 10.1002/jgm.2717

[49] Pan Y, Wang D, Liu F. miR-146b suppresses LPS-induced M1 macrophage polarization via inhibiting the FGL2-activated NF-κB/MAPK signaling pathway in inflammatory bowel disease. Clinics (Sao Paulo). 2022;77: 100069. 35749999 10.1016/j.clinsp.2022.100069 PMC9234609

[50] Peng L, et al. Reprogramming macrophage orientation by microRNA 146b targeting transcription factor IRF5. EBioMedicine. 2016;14:83–96. 27825654 10.1016/j.ebiom.2016.10.041 PMC5161420

[51] Dhuppar S, Murugaiyan G. miRNA effects on gut homeostasis: therapeutic implications for inflammatory bowel disease. Trends Immunol. 2022;43(11):917–31. 36220689 10.1016/j.it.2022.09.003 PMC9617792

[52] Wang L, et al. miR-34a is a microRNA safeguard for Citrobacter-induced inflammatory colon oncogenesis. Elife. 2018;7:39479. 10.7554/eLife.39479 PMC6314783 30543324

[53] Takahashi H, et al. TGF-β and retinoic acid induce the microRNA miR-10a, which targets Bcl-6 and constrains the plasticity of helper T cells. Nat Immunol. 2012;13(6):587–95. 22544395 10.1038/ni.2286 PMC3499969

[54] Yang W, et al. MicroRNA-10a negatively regulates CD4(+) T cell IL-10 production through suppression of blimp1. J Immunol. 2021;207(3):985–95. 34301843 10.4049/jimmunol.2100017 PMC8323958

[55] Ge Y, et al. MicroRNA-125a suppresses intestinal mucosal inflammation through targeting ETS-1 in patients with inflammatory bowel diseases. J Autoimmun. 2019;101:109–20. 31014918 10.1016/j.jaut.2019.04.014

[56] Das LM, et al. TGF-β conditions intestinal T cells to express increased levels of miR-155, associated with down-regulation of IL-2 and itk mRNA. Mucosal Immunol. 2013;6(1):167–76. 22785227 10.1038/mi.2012.60 PMC3504619

[57] Chao G, et al. MiR-155 controls follicular Treg cell-mediated humoral autoimmune intestinal injury by inhibiting CTLA-4 expression. Int Immunopharmacol. 2019;71:267–76. 30927737 10.1016/j.intimp.2019.03.009

[58] Sanctuary MR, et al. miR-106a deficiency attenuates inflammation in murine IBD models. Mucosal Immunol. 2019;12(1):200–11. 30327532 10.1038/s41385-018-0091-7 PMC6301105

[59] Mikami Y, et al. MicroRNA-221 and -222 modulate intestinal inflammatory Th17 cell response as negative feedback regulators downstream of interleukin-23. Immunity. 2021;54(3):514-525.e6. 33657395 10.1016/j.immuni.2021.02.015 PMC8025838

[60] Malumbres R, et al. Differentiation stage-specific expression of microRNAs in B lymphocytes and diffuse large B-cell lymphomas. Blood. 2009;113(16):3754–64. 19047678 10.1182/blood-2008-10-184077 PMC2670792

[61] Zhou B, et al. miR-150, a microRNA expressed in mature B and T cells, blocks early B cell development when expressed prematurely. Proc Natl Acad Sci U S A. 2007;104(17):7080–5. 17438277 10.1073/pnas.0702409104 PMC1855395

[62] Vigorito E, et al. microRNA-155 regulates the generation of immunoglobulin class-switched plasma cells. Immunity. 2007;27(6):847–59. 18055230 10.1016/j.immuni.2007.10.009 PMC4135426

[63] Casali P, et al. Epigenetic modulation of class-switch DNA recombination to IgA by miR-146a through downregulation of Smad2, Smad3 and Smad4. Front Immunol. 2021;12: 761450. 34868004 10.3389/fimmu.2021.761450 PMC8635144

[64] Kuballa P, et al. Autophagy and the immune system. Annu Rev Immunol. 2012;30:611–46. 22449030 10.1146/annurev-immunol-020711-074948

[65] Netea-Maier RT, et al. Modulation of inflammation by autophagy: consequences for human disease. Autophagy. 2016;12(2):245–60. 26222012 10.1080/15548627.2015.1071759 PMC4836004

[66] Mizoguchi A, Mizoguchi E. Inflammatory bowel disease, past, present and future: lessons from animal models. J Gastroenterol. 2008;43(1):1–17. 18297430 10.1007/s00535-007-2111-3

[67] Lapaquette P, Bringer MA, Darfeuille-Michaud A. Defects in autophagy favour adherent-invasive Escherichia coli persistence within macrophages leading to increased pro-inflammatory response. Cell Microbiol. 2012;14(6):791–807. 22309232 10.1111/j.1462-5822.2012.01768.x

[68] Lapaquette P, et al. Crohn’s disease-associated adherent-invasive E. coli are selectively favoured by impaired autophagy to replicate intracellularly. Cell Microbiol. 2010;12(1):99–113. 19747213 10.1111/j.1462-5822.2009.01381.x PMC3743084

[69] Cao B, et al. Role of MiRNAs in inflammatory bowel disease. Dig Dis Sci. 2017;62(6):1426–38. 28391412 10.1007/s10620-017-4567-1

[70] Stiegeler S, et al. The impact of MicroRNAs during inflammatory bowel disease: effects on the mucus layer and intercellular junctions for gut permeability. Cells. 2021;10(12):3358. 34943865 10.3390/cells10123358 PMC8699384

[71] Murphy SF, Kwon JH, Boone DL. Novel players in inflammatory bowel disease pathogenesis. Curr Gastroenterol Rep. 2012;14(2):146–52. 22359107 10.1007/s11894-012-0250-z PMC3324110

[72] Mowat AM, Agace WW. Regional specialization within the intestinal immune system. Nat Rev Immunol. 2014;14(10):667–85. 25234148 10.1038/nri3738

[73] Burgueño JF, Abreu MT. Epithelial Toll-like receptors and their role in gut homeostasis and disease. Nat Rev Gastroenterol Hepatol. 2020;17(5):263–78. 32103203 10.1038/s41575-019-0261-4

[74] Allaire JM, et al. The intestinal epithelium: central coordinator of mucosal immunity. Trends Immunol. 2018;39(9):677–96. 29716793 10.1016/j.it.2018.04.002

[75] Mei X, Gu M, Li M. Plasticity of Paneth cells and their ability to regulate intestinal stem cells. Stem Cell Res Ther. 2020;11(1):349. 32787930 10.1186/s13287-020-01857-7 PMC7425583

[76] Goga A, et al. miR-802 regulates Paneth cell function and enterocyte differentiation in the mouse small intestine. Nat Commun. 2021;12(1):3339. 34099655 10.1038/s41467-021-23298-3 PMC8184787

[77] Kwon MS, et al. MicroRNA-195 regulates Tuft cell function in the intestinal epithelium by altering translation of DCLK1. Am J Physiol Cell Physiol. 2021;320(6):C1042–54. 33788631 10.1152/ajpcell.00597.2020 PMC8285635

[78] Biton M, et al. Epithelial microRNAs regulate gut mucosal immunity via epithelium-T cell crosstalk. Nat Immunol. 2011;12(3):239–46. 21278735 10.1038/ni.1994

[79] Tian Y, et al. MicroRNA-31 reduces inflammatory signaling and promotes regeneration in colon epithelium, and delivery of mimics in microspheres reduces colitis in mice. Gastroenterology. 2019;156(8):2281-2296.e6. 30779922 10.1053/j.gastro.2019.02.023

[80] Wei X, et al. MicroRNA-200 loaded lipid nanoparticles promote intestinal epithelium regeneration in canonical MicroRNA-deficient mice. ACS Nano. 2023;17(22):22901–15. 37939210 10.1021/acsnano.3c08030 PMC10690841

[81] Liu L, et al. miR-381-3p knockdown improves intestinal epithelial proliferation and barrier function after intestinal ischemia/reperfusion injury by targeting nurr1. Cell Death Dis. 2018;9(3):411. 29540663 10.1038/s41419-018-0450-z PMC5852084

[82] Chen W, et al. miR-185-5p / ATG101 axis alleviated intestinal barrier damage in intestinal ischemia reperfusion through autophagy. Heliyon. 2023;9(7): e18325. 37539299 10.1016/j.heliyon.2023.e18325 PMC10395547

[83] Shen L, et al. Tight junction pore and leak pathways: a dynamic duo. Annu Rev Physiol. 2011;73:283–309. 20936941 10.1146/annurev-physiol-012110-142150 PMC4655434

[84] Li M, et al. Mast cells-derived MiR-223 destroys intestinal barrier function by inhibition of CLDN8 expression in intestinal epithelial cells. Biol Res. 2020;53(1):12. 32209121 10.1186/s40659-020-00279-2 PMC7092522

[85] Wang X, et al. MicroRNA-155-5p is a key regulator of allergic inflammation, modulating the epithelial barrier by targeting PKIα. Cell Death Dis. 2019;10(12):884. 31767859 10.1038/s41419-019-2124-x PMC6877533

[86] Martínez C, et al. miR-16 and miR-125b are involved in barrier function dysregulation through the modulation of claudin-2 and cingulin expression in the jejunum in IBS with diarrhoea. Gut. 2017;66(9):1537–8. 28082316 10.1136/gutjnl-2016-311477 PMC5561373

[87] Cordes F, et al. MicroRNA-320a strengthens intestinal barrier function and follows the course of experimental colitis. Inflamm Bowel Dis. 2016;22(10):2341–55. 27607334 10.1097/MIB.0000000000000917

[88] Wang M, et al. IL-21 mediates microRNA-423-5p /claudin-5 signal pathway and intestinal barrier function in inflammatory bowel disease. Aging (Albany NY). 2020;12(16):16099–110. 32855360 10.18632/aging.103566 PMC7485739

[89] Zhuang Y, et al. MicroRNA regulation of endothelial junction proteins and clinical consequence. Mediators Inflamm. 2016;2016:5078627. 27999452 10.1155/2016/5078627 PMC5143735

[90] Ma TY, et al. Mechanism of TNF-{alpha} modulation of Caco-2 intestinal epithelial tight junction barrier: role of myosin light-chain kinase protein expression. Am J Physiol Gastrointest Liver Physiol. 2005;288(3):G422–30. 15701621 10.1152/ajpgi.00412.2004

[91] Wang L, et al. Baicalin protects against TNF-α-induced injury by down-regulating miR-191a that targets the tight junction protein ZO-1 in IEC-6 cells. Biol Pharm Bull. 2017;40(4):435–43. 28111380 10.1248/bpb.b16-00789

[92] Tang Y, et al. The role of miR-212 and iNOS in alcohol-induced intestinal barrier dysfunction and steatohepatitis. Alcohol Clin Exp Res. 2015;39(9):1632–41. 26207424 10.1111/acer.12813 PMC4558329

[93] Zou T, et al. H19 long noncoding RNA Regulates intestinal epithelial barrier function via MicroRNA 675 by interacting with RNA-binding protein HuR. Mol Cell Biol. 2016;36(9):1332–41. 26884465 10.1128/MCB.01030-15 PMC4836219

[94] Zhi X, et al. MiR-874 promotes intestinal barrier dysfunction through targeting AQP3 following intestinal ischemic injury. FEBS Lett. 2014;588(5):757–63. 24462679 10.1016/j.febslet.2014.01.022

[95] Casado-Bedmar M, Viennois E. MicroRNA and gut microbiota: tiny but mighty—novel insights into their cross-talk in inflammatory bowel disease pathogenesis and therapeutics. J Crohns Colitis. 2021;16(6):992–1005. 10.1093/ecco-jcc/jjab223 PMC9282881 34918052

[96] Liu S, et al. The host shapes the gut microbiota via fecal MicroRNA. Cell Host Microbe. 2016;19(1):32–43. 26764595 10.1016/j.chom.2015.12.005 PMC4847146

[97] Dalmasso G, et al. Microbiota modulate host gene expression via microRNAs. PLoS ONE. 2011;6(4): e19293. 21559394 10.1371/journal.pone.0019293 PMC3084815

[98] Nguyen HT, et al. Crohn’s disease-associated adherent invasive Escherichia coli modulate levels of microRNAs in intestinal epithelial cells to reduce autophagy. Gastroenterology. 2014;146(2):508–19. 24148619 10.1053/j.gastro.2013.10.021

[99] Viennois E, et al. Host-derived fecal microRNAs can indicate gut microbiota healthiness and ability to induce inflammation. Theranostics. 2019;9(15):4542–57. 31285778 10.7150/thno.35282 PMC6599659

[100] Tomkovich S, et al. Human colon mucosal biofilms and murine host communicate via altered mRNA and microRNA expression during cancer. mSystems. 2020;5(1):10–1128. 10.1128/msystems.00451-19. 10.1128/mSystems.00451-19 PMC6967385 31937674

[101] Johnston DGW, et al. Loss of MicroRNA-21 Influences the gut microbiota, causing reduced susceptibility in a murine model of colitis. J Crohns Colitis. 2018;12(7):835–48. 29608690 10.1093/ecco-jcc/jjy038

[102] Wu F, et al. MicroRNAs are differentially expressed in ulcerative colitis and alter expression of macrophage inflammatory peptide-2 alpha. Gastroenterology. 2008;135(5):1624-1635.e24. 18835392 10.1053/j.gastro.2008.07.068

[103] Mohammadi A, et al. Differential expression of microRNAs in Peripheral blood mononuclear cells identifies autophagy and TGF-beta-related signatures aberrantly expressed in inflammatory bowel disease. J Crohns Colitis. 2018;12(5):568–81. 29420705 10.1093/ecco-jcc/jjy010 PMC6018685

[104] Masi L, et al. MicroRNAs as innovative biomarkers for inflammatory bowel disease and prediction of colorectal cancer. Int J Mol Sci. 2022;23(14):7991. 35887337 10.3390/ijms23147991 PMC9318064

[105] Wu F, et al. Identification of microRNAs associated with ileal and colonic Crohn’s disease. Inflamm Bowel Dis. 2010;16(10):1729–38. 20848482 10.1002/ibd.21267 PMC2946509

[106] Schönauen K, et al. Circulating and fecal microRNAs as biomarkers for inflammatory bowel diseases. Inflamm Bowel Dis. 2018;24(7):1547–57. 29668922 10.1093/ibd/izy046

[107] Suri K, et al. Role of MicroRNA in inflammatory bowel disease: clinical evidence and the development of preclinical animal models. Cells. 2021;10(9):2204. 34571853 10.3390/cells10092204 PMC8468560

[108] Schaefer JS, et al. MicroRNA signatures differentiate Crohn’s disease from ulcerative colitis. BMC Immunol. 2015;16:5. 25886994 10.1186/s12865-015-0069-0 PMC4335694

[109] Yarani R, et al. Differentially expressed miRNAs in ulcerative colitis and Crohn’s disease. Front Immunol. 2022;13:865777. 35734163 10.3389/fimmu.2022.865777 PMC9208551

[110] Shannon P, et al. Cytoscape: a software environment for integrated models of biomolecular interaction networks. Genome Res. 2003;13(11):2498–504. 14597658 10.1101/gr.1239303 PMC403769

[111] Krämer A, et al. Causal analysis approaches in ingenuity pathway analysis. Bioinformatics. 2014;30(4):523–30. 24336805 10.1093/bioinformatics/btt703 PMC3928520

[112] Wang X, et al. MicroRNA Let-7i negatively regulates cardiac inflammation and fibrosis. Hypertension. 2015;66(4):776–85. 26259595 10.1161/HYPERTENSIONAHA.115.05548

[113] Satoh M, et al. Expression of let-7i is associated with Toll-like receptor 4 signal in coronary artery disease: effect of statins on let-7i and Toll-like receptor 4 signal. Immunobiology. 2012;217(5):533–9. 21899916 10.1016/j.imbio.2011.08.005

[114] Zhai Y, et al. Coordinated changes in mRNA turnover, translation, and RNA processing bodies in bronchial epithelial cells following inflammatory stimulation. Mol Cell Biol. 2008;28(24):7414–26. 18936174 10.1128/MCB.01237-08 PMC2593439

[115] Tian T, et al. MicroRNA-16 is putatively involved in the NF-κB pathway regulation in ulcerative colitis through adenosine A2a receptor (A2aAR) mRNA targeting. Sci Rep. 2016;6:30824. 27476546 10.1038/srep30824 PMC4967855

[116] Lewis A, et al. Low serum levels of MicroRNA-19 are associated with a stricturing Crohn’s disease phenotype. Inflamm Bowel Dis. 2015;21(8):1926–34. 25985247 10.1097/MIB.0000000000000443

[117] Collins AS, et al. miR-19a: an effective regulator of SOCS3 and enhancer of JAK-STAT signalling. PLoS ONE. 2013;8(7): e69090. 23894411 10.1371/journal.pone.0069090 PMC3718810

[118] Sandborn WJ, et al. Ustekinumab induction and maintenance therapy in refractory Crohn’s disease. N Engl J Med. 2012;367(16):1519–28. 23075178 10.1056/NEJMoa1203572

[119] Wu H, et al. miRNA profiling of naïve, effector and memory CD8 T cells. PLoS ONE. 2007;2(10): e1020. 17925868 10.1371/journal.pone.0001020 PMC2000354

[120] Yang Y, et al. Overexpression of miR-21 in patients with ulcerative colitis impairs intestinal epithelial barrier function through targeting the Rho GTPase RhoB. Biochem Biophys Res Commun. 2013;434(4):746–52. 23583411 10.1016/j.bbrc.2013.03.122

[121] Zhou R, et al. Identification of microRNA-16-5p and microRNA-21-5p in feces as potential noninvasive biomarkers for inflammatory bowel disease. Aging (Albany NY). 2021;13(3):4634–46. 33535181 10.18632/aging.202428 PMC7906140

[122] Yan H, Zhang X, Xu Y. Aberrant expression of miR-21 in patients with inflammatory bowel disease: A protocol for systematic review and meta analysis. Medicine (Baltimore). 2020;99(17): e19693. 32332611 10.1097/MD.0000000000019693 PMC7220677

[123] Momen-Heravi F, Bala S. miRNA regulation of innate immunity. J Leukoc Biol. 2018;103(6):1205–17. 10.1002/JLB.3MIR1117-459R 29656417

[124] Yang G, et al. Discovery and validation of extracellular/circulating microRNAs during idiopathic pulmonary fibrosis disease progression. Gene. 2015;562(1):138–44. 25725128 10.1016/j.gene.2015.02.065

[125] Zhao J, et al. MiR-21 simultaneously regulates ERK1 signaling in HSC activation and hepatocyte EMT in hepatic fibrosis. PLoS ONE. 2014;9(10): e108005. 25303175 10.1371/journal.pone.0108005 PMC4193742

[126] Ye SB, et al. Exosomal miR-24-3p impedes T-cell function by targeting FGF11 and serves as a potential prognostic biomarker for nasopharyngeal carcinoma. J Pathol. 2016;240(3):329–40. 27538493 10.1002/path.4781

[127] Fiedler J, et al. MicroRNA-24 regulates vascularity after myocardial infarction. Circulation. 2011;124(6):720–30. 21788589 10.1161/CIRCULATIONAHA.111.039008

[128] Yu T, et al. Long noncoding RNA PMS2L2 downregulates miR-24 through methylation to suppress cell apoptosis in ulcerative colitis. Dig Dis. 2021;39(5):467–76. 33238281 10.1159/000513330

[129] Luna C, et al. MicroRNA-24 regulates the processing of latent TGFβ1 during cyclic mechanical stress in human trabecular meshwork cells through direct targeting of FURIN. J Cell Physiol. 2011;226(5):1407–14. 20945401 10.1002/jcp.22476 PMC3152464

[130] Lv B, et al. MiR-29a promotes intestinal epithelial apoptosis in ulcerative colitis by down-regulating Mcl-1. Int J Clin Exp Pathol. 2014;7(12):8542–52. 25674218 PMC4313986

[131] Chen T, et al. MicroRNA-29a regulates pro-inflammatory cytokine secretion and scavenger receptor expression by targeting LPL in oxLDL-stimulated dendritic cells. FEBS Lett. 2011;585(4):657–63. 21276447 10.1016/j.febslet.2011.01.027

[132] Ghadiri N, et al. Analysis of the expression of mir-34a, mir-199a, mir-30c and mir-19a in peripheral blood CD4+T lymphocytes of relapsing-remitting multiple sclerosis patients. Gene. 2018;659:109–17. 29551498 10.1016/j.gene.2018.03.035

[133] Olaru AV, et al. Dynamic changes in the expression of MicroRNA-31 during inflammatory bowel disease-associated neoplastic transformation. Inflamm Bowel Dis. 2011;17(1):221–31. 20848542 10.1002/ibd.21359 PMC3006011

[134] Xu N, et al. MicroRNA-31 is overexpressed in psoriasis and modulates inflammatory cytokine and chemokine production in keratinocytes via targeting serine/threonine kinase 40. J Immunol. 2013;190(2):678–88. 23233723 10.4049/jimmunol.1202695

[135] Zhang L, et al. MicroRNA-31 negatively regulates peripherally derived regulatory T-cell generation by repressing retinoic acid-inducible protein 3. Nat Commun. 2015;6:7639. 26165721 10.1038/ncomms8639 PMC4510656

[136] Gwiggner M, et al. MicroRNA-31 and MicroRNA-155 are overexpressed in ulcerative colitis and regulate IL-13 signaling by targeting interleukin 13 receptor α-1. Genes (Basel). 2018;9(2):85. 29438285 10.3390/genes9020085 PMC5852581

[137] Jovov B, et al. Defective barrier function in neosquamous epithelium. Am J Gastroenterol. 2013;108(3):386–91. 23318477 10.1038/ajg.2012.440 PMC3838860

[138] Qu J, et al. The spring-like effect of microRNA-31 in balancing inflammatory and regenerative responses in colitis. Front Microbiol. 2022;13:1089729. 36590397 10.3389/fmicb.2022.1089729 PMC9800619

[139] Omidbakhsh A, et al. Micro-RNAs-106a and -362-3p in peripheral blood of inflammatory bowel disease patients. Open Biochem J. 2018;12:78–86. 30069249 10.2174/1874091X01812010078 PMC6040212

[140] Bronevetsky Y, et al. T cell activation induces proteasomal degradation of Argonaute and rapid remodeling of the microRNA repertoire. J Exp Med. 2013;210(2):417–32. 23382546 10.1084/jem.20111717 PMC3570096

[141] Zhu D, et al. MicroRNA-17/20a/106a modulate macrophage inflammatory responses through targeting signal-regulatory protein α. J Allergy Clin Immunol. 2013;132(2):426-36.e8. 23562609 10.1016/j.jaci.2013.02.005 PMC5882493

[142] Li D, et al. MiRNA-374b-5p and miRNA-106a-5p are related to inflammatory bowel disease via regulating IL-10 and STAT3 signaling pathways. BMC Gastroenterol. 2022;22(1):492. 36437465 10.1186/s12876-022-02533-1 PMC9703806

[143] El Sabbagh E, et al. Role of circulating microRNA146b-5p and microRNA-106a in diagnosing and predicting the severity of inflammatory bowel disease. Egypt J Chem. 2023;66(12):545–51.

[144] Sharma A, et al. Posttranscriptional regulation of interleukin-10 expression by hsa-miR-106a. Proc Natl Acad Sci U S A. 2009;106(14):5761–6. 19307576 10.1073/pnas.0808743106 PMC2659714

[145] Anandagoda N, et al. microRNA-142-mediated repression of phosphodiesterase 3B critically regulates peripheral immune tolerance. J Clin Invest. 2019;129(3):1257–71. 30741720 10.1172/JCI124725 PMC6391082

[146] Amato G, et al. Involvement of miR-142 and miR-155 in non-infectious complications of CVID. Molecules. 2020;25(20):4760. 33081305 10.3390/molecules25204760 PMC7587593

[147] Han J, et al. lncRNA TUG1 regulates ulcerative colitis through miR-142-5p/SOCS1 axis. Microb Pathog. 2020;143: 104139. 32173492 10.1016/j.micpath.2020.104139

[148] Xiang P, et al. Protective role of circRNA CCND1 in ulcerative colitis via miR-142-5p/NCOA3 axis. BMC Gastroenterol. 2023;23(1):18. 36658474 10.1186/s12876-023-02641-6 PMC9850594

[149] Han J, et al. MicroRNA-142-5p facilitates the pathogenesis of ulcerative colitis by regulating SOCS1. Int J Clin Exp Pathol. 2018;11(12):5735–44. 31949659 PMC6963094

[150] Duijvis NW, et al. Inhibition of miR-142-5P ameliorates disease in mouse models of experimental colitis. PLoS ONE. 2017;12(10): e0185097. 29059189 10.1371/journal.pone.0185097 PMC5653202

[151] Tili E, et al. MicroRNAs in intestinal barrier function, inflammatory bowel disease and related cancers—their effects and therapeutic potentials. Curr Opin Pharmacol. 2017;37:142–50. 29154194 10.1016/j.coph.2017.10.010 PMC5938753

[152] Li G, et al. miR-142-3p encapsulated in T lymphocyte-derived tissue small extracellular vesicles induces Treg function defect and thyrocyte destruction in Hashimoto’s thyroiditis. BMC Med. 2023;21(1):206. 37280674 10.1186/s12916-023-02914-7 PMC10242775

[153] Gao J, et al. Blockade of miR-142-3p promotes anti-apoptotic and suppressive function by inducing KDM6A-mediated H3K27me3 demethylation in induced regulatory T cells. Cell Death Dis. 2019;10(5):332. 30988391 10.1038/s41419-019-1565-6 PMC6465300

[154] Bian Z, et al. Role of miR-150-targeting c-Myb in colonic epithelial disruption during dextran sulphate sodium-induced murine experimental colitis and human ulcerative colitis. J Pathol. 2011;225(4):544–53. 21590770 10.1002/path.2907

[155] Ishihara S, et al. Deletion of miR-150 Prevents Spontaneous T Cell Proliferation and the Development of Colitis. Gastro Hep Advances. 2023;2(4):487–96. 39132043 10.1016/j.gastha.2023.01.021 PMC11308117

[156] Plank M, et al. Targeting translational control as a novel way to treat inflammatory disease: the emerging role of microRNAs. Clin Exp Allergy. 2013;43(9):981–99. 23957346 10.1111/cea.12170

[157] Rodríguez-Nogales A, et al. Differential intestinal anti-inflammatory effects of Lactobacillus fermentum and Lactobacillus salivarius in DSS mouse colitis: impact on microRNAs expression and microbiota composition. Mol Nutr Food Res. 2017;61(11):1700144. 10.1002/mnfr.201700144 28752563

[158] Wan J, et al. Expression and function of miR-155 in diseases of the gastrointestinal tract. Int J Mol Sci. 2016;17(5):709. 27187359 10.3390/ijms17050709 PMC4881531

[159] Xiao X, et al. miRNAs can affect intestinal epithelial barrier in inflammatory bowel disease. Front Immunol. 2022;13:868229. 35493445 10.3389/fimmu.2022.868229 PMC9043318

[160] Evel-Kabler K, et al. SOCS1 restricts dendritic cells’ ability to break self tolerance and induce antitumor immunity by regulating IL-12 production and signaling. J Clin Invest. 2006;116(1):90–100. 16357940 10.1172/JCI26169 PMC1312019

[161] Zhu F, et al. miR-155 antagomir protect against DSS-induced colitis in mice through regulating Th17/Treg cell balance by Jarid2/Wnt/β-catenin. Biomed Pharmacother. 2020;126: 109909. 32135463 10.1016/j.biopha.2020.109909

[162] Wu F, et al. Peripheral blood microRNAs distinguish active ulcerative colitis and Crohn’s disease. Inflamm Bowel Dis. 2011;17(1):241–50. 20812331 10.1002/ibd.21450 PMC2998576

[163] Yang L, et al. Hsa_circ_0060450 negatively regulates type I interferon-induced inflammation by serving as miR-199a-5p sponge in type 1 diabetes mellitus. Front Immunol. 2020;11:576903. 33133095 10.3389/fimmu.2020.576903 PMC7550460

[164] Wang S, et al. Suppression of miR-199a-5p alleviates ulcerative colitis by upregulating endoplasmic reticulum stress component XBP1. bioRxiv. 2021;2021:02.

[165] Verdier J, et al. Faecal micro-RNAs in inflammatory bowel diseases. J Crohns Colitis. 2020;14(1):110–7. 31209454 10.1093/ecco-jcc/jjz120

[166] Wohnhaas CT, et al. Fecal MicroRNAs Show promise as noninvasive Crohn’s disease biomarkers. Crohns Colitis 360. 2020;2(1):otaa003. 32551441 10.1093/crocol/otaa003 PMC7291945

[167] Zhang J, et al. miR-223 improves intestinal inflammation through inhibiting the IL-6/STAT3 signaling pathway in dextran sodium sulfate-induced experimental colitis. Immun, Inflamm Dis. 2021;9(1):319–27. 33332758 10.1002/iid3.395 PMC7860526

[168] Rosa A, et al. The interplay between the master transcription factor PU.1 and miR-424 regulates human monocyte/macrophage differentiation. Proc Natl Acad Sci USA. 2007;104(50):19849–54. 18056638 10.1073/pnas.0706963104 PMC2148386

[169] Coll S, et al. P020 differential expression of miR-424–5p and miR-378c in stenotic and penetrating lessions of Crohn’s disease associates with altered transcription of genes involved in vascular regulation. J Crohn’s Colitis. 2022;16:i146–7.

[170] Dai W, et al. miR-424-5p promotes the proliferation and metastasis of colorectal cancer by directly targeting SCN4B. Pathol—Res Pract. 2020;216(1): 152731. 31785995 10.1016/j.prp.2019.152731

[171] Zhu S, et al. The microRNA miR-23b suppresses IL-17-associated autoimmune inflammation by targeting TAB2, TAB3 and IKK-α. Nat Med. 2012;18(7):1077–86. 22660635 10.1038/nm.2815

[172] Lv Y, et al. Strand-specific miR-28-3p and miR-28-5p have differential effects on nasopharyngeal cancer cells proliferation, apoptosis, migration and invasion. Cancer Cell Int. 2019;19:187. 31360121 10.1186/s12935-019-0915-x PMC6642532

[173] Li Q, et al. miR-28 modulates exhaustive differentiation of T cells through silencing programmed cell death-1 and regulating cytokine secretion. Oncotarget. 2016;7(33):53735–50. 27447564 10.18632/oncotarget.10731 PMC5288217

[174] Liu S, et al. Oral Administration of miR-30d from Feces of MS patients suppresses MS-like symptoms in Mice by expanding akkermansia muciniphila. Cell Host Microbe. 2019;26(6):779-794.e8. 31784260 10.1016/j.chom.2019.10.008 PMC6948921

[175] Feng X, et al. Up-regulation of microRNA-126 may contribute to pathogenesis of ulcerative colitis via regulating NF-kappaB inhibitor IκBα. PLoS ONE. 2012;7(12): e52782. 23285182 10.1371/journal.pone.0052782 PMC3532399

[176] Harris TA, et al. MicroRNA-126 regulates endothelial expression of vascular cell adhesion molecule 1. Proc Natl Acad Sci U S A. 2008;105(5):1516–21. 18227515 10.1073/pnas.0707493105 PMC2234176

[177] Chu X, et al. Downregulation of miR-126-3p expression contributes to increased inflammatory response in placental trophoblasts in preeclampsia. J Reprod Immunol. 2021;144: 103281. 33549904 10.1016/j.jri.2021.103281

[178] Zou Q, et al. miR-126 ameliorates multiple organ dysfunction in septic rats by regulating the differentiation of Th17/Treg. Mol Biol Rep. 2022;49(4):2985–98. 35122598 10.1007/s11033-022-07121-w PMC8817156

[179] Pan J, et al. MicroRNA-126-3p/-5p overexpression attenuates blood-brain barrier disruption in a mouse model of middle cerebral artery occlusion. Stroke. 2020;51(2):619–27. 31822249 10.1161/STROKEAHA.119.027531

[180] Jiang L, et al. microRNA-126 inhibits vascular cell adhesion molecule-1 and interleukin-1beta in human dental pulp cells. J Clin Lab Anal. 2022;36(5): e24371. 35334501 10.1002/jcla.24371 PMC9102615

[181] Kim EH, Suresh M. Role of PI3K/Akt signaling in memory CD8 T cell differentiation. Front Immunol. 2013;4:20. 23378844 10.3389/fimmu.2013.00020 PMC3561661

[182] Li H, et al. MiR-140-5p inhibits synovial fibroblasts proliferation and inflammatory cytokines secretion through targeting TLR4. Biomed Pharmacother. 2017;96:208–14. 28987944 10.1016/j.biopha.2017.09.079

[183] Su J, et al. MicroRNA-140–5p ameliorates the high glucose-induced apoptosis and inflammation through suppressing TLR4/NF-κB signaling pathway in human renal tubular epithelial cells. Biosci Rep. 2020;40(3):BSR20192384. 32073611 10.1042/BSR20192384 PMC7056448

[184] Ghafouri-Fard S, et al. microRNA-140: A miRNA with diverse roles in human diseases. Biomed Pharmacother. 2021;135: 111256. 33434855 10.1016/j.biopha.2021.111256

[185] Zhang Q, et al. Overexpression of miR-140-5p inhibits lipopolysaccharide-induced human intervertebral disc inflammation and degeneration by downregulating toll-like receptor 4. Oncol Rep. 2018;40(2):793–802. 29901170 10.3892/or.2018.6488

[186] Zhu J, et al. MicroRNA-140-5p regulates the proliferation, apoptosis and inflammation of RA FLSs by repressing STAT3. Exp Ther Med. 2021;21(2):171. 33456538 10.3892/etm.2020.9602 PMC7792473

[187] Yang Y, et al. Upregulation of miRNA-140-5p inhibits inflammatory cytokines in acute lung injury through the MyD88/NF-κB signaling pathway by targeting TLR4. Exp Ther Med. 2018;16(5):3913–20. 30344669 10.3892/etm.2018.6692 PMC6176196

[188] Joyce CE, et al. Deep sequencing of small RNAs from human skin reveals major alterations in the psoriasis miRNAome. Hum Mol Genet. 2011;20(20):4025–40. 21807764 10.1093/hmg/ddr331 PMC3177648

[189] Chan EK, Satoh M, Pauley KM. Contrast in aberrant microRNA expression in systemic lupus erythematosus and rheumatoid arthritis: is microRNA-146 all we need? Arthritis Rheum. 2009;60(4):912–5. 19333929 10.1002/art.24421 PMC2792591

[190] Huang Z, et al. miR-141 regulates colonic leukocytic trafficking by targeting CXCL12β during murine colitis and human Crohn’s disease. Gut. 2014;63(8):1247–57. 24000293 10.1136/gutjnl-2012-304213

[191] Pan A, et al. STAT4 silencing underlies a novel inhibitory role of microRNA-141-3p in inflammation response of mice with experimental autoimmune myocarditis. Am J Physiol Heart Circ Physiol. 2019;317(3):H531–40. 31225989 10.1152/ajpheart.00048.2019

[192] Shen WS, et al. Potential mechanisms of microRNA-141-3p to alleviate chronic inflammatory pain by downregulation of downstream target gene HMGB1: in vitro and in vivo studies. Gene Ther. 2017;24(6):353–60. 28440797 10.1038/gt.2017.28

[193] Chen H, et al. Increased expression of microRNA-141–3p improves necrotizing enterocolitis of neonates through targeting MNX1. Front Pediat. 2020. 10.3389/fped.2020.00385. 10.3389/fped.2020.00385 PMC7399201 32850524

[194] Li X, et al. MiR-141-3p ameliorates RIPK1-mediated necroptosis of intestinal epithelial cells in necrotizing enterocolitis. Aging (Albany NY). 2020;12(18):18073–83. 32702669 10.18632/aging.103608 PMC7585103

[195] Yan R, Liang X, Hu J. miR-141-3p alleviates ulcerative colitis by targeting SUGT1 to inhibit colonic epithelial cell pyroptosis. Autoimmunity. 2023;56(1):2220988. 37317573 10.1080/08916934.2023.2220988

[196] Sonkoly E, Ståhle M, Pivarcsi A. MicroRNAs: novel regulators in skin inflammation. Clin Exp Dermatol. 2008;33(3):312–5. 18419608 10.1111/j.1365-2230.2008.02804.x

[197] Boldin MP, et al. miR-146a is a significant brake on autoimmunity, myeloproliferation, and cancer in mice. J Exp Med. 2011;208(6):1189–201. 21555486 10.1084/jem.20101823 PMC3173243

[198] Ghorpade DS, et al. NOD2-nitric oxide-responsive microRNA-146a activates Sonic hedgehog signaling to orchestrate inflammatory responses in murine model of inflammatory bowel disease. J Biol Chem. 2013;288(46):33037–48. 24092752 10.1074/jbc.M113.492496 PMC3829153

[199] Taganov KD, et al. NF-κB-dependent induction of microRNA miR-146, an inhibitor targeted to signaling proteins of innate immune responses. Proc Natl Acad Sci. 2006;103(33):12481–6. 16885212 10.1073/pnas.0605298103 PMC1567904

[200] Li S, et al. MicroRNA-146a represses mycobacteria-induced inflammatory response and facilitates bacterial replication via targeting IRAK-1 and TRAF-6. PLoS ONE. 2013;8(12): e81438. 24358114 10.1371/journal.pone.0081438 PMC3864784

[201] Li Y, et al. miR-146a-5p negatively regulates the IL-1β-stimulated inflammatory response via downregulation of the IRAK1/TRAF6 signaling pathway in human intestinal epithelial cells. Exp Ther Med. 2022;24(4):615. 36160881 10.3892/etm.2022.11552 PMC9468834

[202] Garo LP, et al. MicroRNA-146a limits tumorigenic inflammation in colorectal cancer. Nat Commun. 2021;12(1):2419. 33893298 10.1038/s41467-021-22641-y PMC8065171

[203] O’Connell RM, et al. Physiological and pathological roles for microRNAs in the immune system. Nat Rev Immunol. 2010;10(2):111–22. 20098459 10.1038/nri2708

[204] Fulzele S, et al. MicroRNA-146b-3p regulates retinal inflammation by suppressing adenosine deaminase-2 in diabetes. Biomed Res Int. 2015;2015: 846501. 25815338 10.1155/2015/846501 PMC4359882

[205] Cai F, et al. MicroRNA-146b-3p regulates the development and progression of cerebral infarction with diabetes through RAF1/P38MAPK/COX-2 signaling pathway. Am J Transl Res. 2018;10(2):618–28. 29511456 PMC5835827

[206] Luo S, Chen XH. Tissue and serum miR-149–3p/5p in hospitalized patients with inflammatory bowel disease: correlation with disease severity and inflammatory markers. Kaohsiung J Med Sci. 2023;40:131–8. 37997516 10.1002/kjm2.12784 PMC11895588

[207] Cao Y, et al. Enterotoxigenic bacteroidesfragilis promotes intestinal inflammation and malignancy by inhibiting exosome-packaged miR-149-3p. Gastroenterology. 2021;161(5):1552-1566.e12. 34371001 10.1053/j.gastro.2021.08.003

[208] Feng Q, et al. Deficiency of miRNA-149-3p shaped gut microbiota and enhanced dextran sulfate sodium-induced colitis. Mol Ther Nucleic Acids. 2022;30:208–25. 36250208 10.1016/j.omtn.2022.09.018 PMC9556934

[209] Ahmed FE, et al. Diagnostic microRNA markers for screening sporadic human colon cancer and active ulcerative colitis in stool and tissue. Cancer Genomics Proteomics. 2009;6(5):281–95. 19996134

[210] Chuang AY, et al. NOD2 expression is regulated by microRNAs in colonic epithelial HCT116 cells. Inflamm Bowel Dis. 2014;20(1):126–35. 24297055 10.1097/01.MIB.0000436954.70596.9b PMC4965169

[211] Bras JP, et al. miR-195 inhibits macrophages pro-inflammatory profile and impacts the crosstalk with smooth muscle cells. PLoS ONE. 2017;12(11): e0188530. 29166412 10.1371/journal.pone.0188530 PMC5699821

[212] Zidar N, et al. Down-regulation of microRNAs of the miR-200 family and up-regulation of Snail and Slug in inflammatory bowel diseases - hallmark of epithelial-mesenchymal transition. J Cell Mol Med. 2016;20(10):1813–20. 27113480 10.1111/jcmm.12869 PMC5020622

[213] Chen Y, et al. miR-200b inhibits TGF-β1-induced epithelial-mesenchymal transition and promotes growth of intestinal epithelial cells. Cell Death Dis. 2013;4(3): e541. 23492772 10.1038/cddis.2013.22 PMC3613822

[214] Yang J, et al. miR-200b-containing microvesicles attenuate experimental colitis associated intestinal fibrosis by inhibiting epithelial-mesenchymal transition. J Gastroenterol Hepatol. 2017;32(12):1966–74. 28370348 10.1111/jgh.13797

[215] Mehta SJ, et al. Epithelial down-regulation of the miR-200 family in fibrostenosing Crohn’s disease is associated with features of epithelial to mesenchymal transition. J Cell Mol Med. 2018;22(11):5617–28. 30188001 10.1111/jcmm.13836 PMC6201355

[216] Xiao Y, et al. Clostridium butyricum partially regulates the development of colitis-associated cancer through miR-200c. Cell Mol Biol (Noisy-le-grand). 2017;63(4):59–66. 28478805 10.14715/cmb/2017.63.4.10

[217] Shen Y, et al. miR-200b inhibits TNF-α-induced IL-8 secretion and tight junction disruption of intestinal epithelial cells in vitro. Am J Physiol Gastrointest Liver Physiol. 2017;312(2):G123–32. 27979826 10.1152/ajpgi.00316.2016

[218] Wendlandt EB, et al. The role of microRNAs miR-200b and miR-200c in TLR4 signaling and NF-κB activation. Innate Immun. 2012;18(6):846–55. 22522429 10.1177/1753425912443903 PMC3733339

[219] Alam KJ, et al. MicroRNA 375 regulates proliferation and migration of colon cancer cells by suppressing the CTGF-EGFR signaling pathway. Int J Cancer. 2017;141(8):1614–29. 28670764 10.1002/ijc.30861

[220] Cheng S, et al. MiR-375-3p alleviates the severity of inflammation through targeting YAP1/LEKTI pathway in HaCaT cells. Biosci Biotechnol Biochem. 2020;84(10):2005–13. 32564679 10.1080/09168451.2020.1783196

[221] Dubois-Camacho K, et al. Inhibition of miR-378a-3p by inflammation enhances IL-33 levels: a novel mechanism of alarmin modulation in ulcerative colitis. Front Immunol. 2019;10:2449. 31824476 10.3389/fimmu.2019.02449 PMC6879552

[222] Li P, et al. Mesenchymal stem cells-derived extracellular vesicles containing miR-378a-3p inhibit the occurrence of inflammatory bowel disease by targeting GATA2. J Cell Mol Med. 2022;26(11):3133–46. 35582765 10.1111/jcmm.17176 PMC9170824

[223] Bellafante E, et al. PGC-1β promotes enterocyte lifespan and tumorigenesis in the intestine. Proc Natl Acad Sci U S A. 2014;111(42):E4523–31. 25288742 10.1073/pnas.1415279111 PMC4210309

[224] Liu C, Lin JD. PGC-1 coactivators in the control of energy metabolism. Acta Biochim Biophys Sin (Shanghai). 2011;43(4):248–57. 21325336 10.1093/abbs/gmr007 PMC3063079

[225] Carrer M, et al. Control of mitochondrial metabolism and systemic energy homeostasis by microRNAs 378 and 378*. Proc Natl Acad Sci U S A. 2012;109(38):15330–5. 22949648 10.1073/pnas.1207605109 PMC3458360

[226] Sifroni KG, et al. Mitochondrial respiratory chain in the colonic mucosal of patients with ulcerative colitis. Mol Cell Biochem. 2010;342(1–2):111–5. 20440543 10.1007/s11010-010-0474-x

[227] Dinesh P, et al. MicroRNA-532-3p regulates pro-inflammatory human THP-1 macrophages by targeting ASK1/p38 MAPK pathway. Inflammation. 2021;44(1):229–42. 32876895 10.1007/s10753-020-01325-7

[228] Innocenti T, et al. MiRNA-Based therapies for the treatment of inflammatory bowel disease: What are we still missing? Inflamm Bowel Dis. 2022;29(2):308–23. 10.1093/ibd/izac122 35749310

[229] Fedyk ER, et al. Exclusive antagonism of the α4 β7 integrin by vedolizumab confirms the gut-selectivity of this pathway in primates. Inflamm Bowel Dis. 2012;18(11):2107–19. 22419649 10.1002/ibd.22940

[230] Pathak S, et al. MiR-155 modulates the inflammatory phenotype of intestinal myofibroblasts by targeting SOCS1 in ulcerative colitis. Exp Mol Med. 2015;47(5): e164. 25998827 10.1038/emm.2015.21 PMC4454995

[231] Herrera-deGuise C, et al. JAK inhibitors: a new dawn for oral therapies in inflammatory bowel diseases. Front Med. 2023;10:1089099. 10.3389/fmed.2023.1089099 PMC10017532 36936239

[232] Lima JF, et al. Anti-miRNA oligonucleotides: a comprehensive guide for design. RNA Biol. 2018;15(3):338–52. 29570036 10.1080/15476286.2018.1445959 PMC5927725

[233] Ye D, et al. MicroRNA regulation of intestinal epithelial tight junction permeability. Gastroenterology. 2011;141(4):1323–33. 21763238 10.1053/j.gastro.2011.07.005 PMC3724217

[234] Yin B, Tian-Chu H, Ling-Fen X. Protection by microRNA-7a-5p antagomir against intestinal mucosal injury related to the JNK pathway in TNBS-induced experimental colitis. Turk J Gastroenterol. 2021;32(5):431–6. 34231472 10.5152/tjg.2021.20746 PMC8975295

[235] Liu Y, et al. MiR-155 contributes to intestinal barrier dysfunction in DSS-induced mice colitis via targeting HIF-1α/TFF-3 axis. Aging (Albany NY). 2020;12(14):14966–77. 32713852 10.18632/aging.103555 PMC7425479

[236] Fang K, et al. MicroRNA-31-3p Is involved in substance P (SP)-associated inflammation in human colonic epithelial cells and experimental colitis. Am J Pathol. 2018;188(3):586–99. 29253460 10.1016/j.ajpath.2017.10.023 PMC5840489

[237] Chen Y, et al. Inhibition of miR-16 ameliorates inflammatory bowel disease by modulating Bcl-2 in mouse models. J Surg Res. 2020;253:185–92. 32361613 10.1016/j.jss.2020.03.037

[238] Valmiki S, et al. miR-125b and miR-223 Contribute to inflammation by targeting the key molecules of NFκB pathway. Front Med (Lausanne). 2019;6:313. 32039213 10.3389/fmed.2019.00313 PMC6990118

[239] Deng F, et al. a molecular targeted immunotherapeutic strategy for ulcerative colitis via dual-targeting nanoparticles delivering miR-146b to intestinal macrophages. J Crohns Colitis. 2018;13(4):482–94. 10.1093/ecco-jcc/jjy181 30445446

[240] Wani S, Man Law IK, Pothoulakis C. Role and mechanisms of exosomal miRNAs in IBD pathophysiology. Am J Physiol Gastrointest Liver Physiol. 2020;319(6):G646–54. 33026230 10.1152/ajpgi.00295.2020 PMC7792667

[241] Wang X, et al. Exosomes as a new delivery vehicle in inflammatory bowel disease. Pharmaceutics. 2021;13(10):1644. 34683937 10.3390/pharmaceutics13101644 PMC8539337

[242] Moon B, Chang S. Exosome as a delivery vehicle for cancer therapy. Cells. 2022;11(3):316. 35159126 10.3390/cells11030316 PMC8834560

[243] Wang C, Chen J. microRNAs as therapeutic targets in intestinal diseases. ExRNA. 2019;1(1):1–12.34171007

[244] Tazi J, et al. Specific and selective induction of miR-124 in immune cells by the quinoline ABX464: a transformative therapy for inflammatory diseases. Drug Discov Today. 2021;26(4):1030–9. 33387693 10.1016/j.drudis.2020.12.019

[245] Vautrin A, et al. Both anti-inflammatory and antiviral properties of novel drug candidate ABX464 are mediated by modulation of RNA splicing. Sci Rep. 2019;9(1):792. 30692590 10.1038/s41598-018-37813-y PMC6349857

[246] Chebli K, et al. The Anti-Hiv candidate Abx464 dampens intestinal inflammation by triggering Il-22 production in activated macrophages. Sci Rep. 2017;7(1):4860. 28687795 10.1038/s41598-017-04071-3 PMC5501810

[247] Vermeire S, et al. Induction and long-term follow-up with ABX464 for Moderate-to-severe ulcerative colitis: results of phase IIa trial. Gastroenterology. 2021;160(7):2595-2598.e3. 33662385 10.1053/j.gastro.2021.02.054

[248] Vermeire S, et al. ABX464 (obefazimod) for moderate-to-severe, active ulcerative colitis: a phase 2b, double-blind, randomised, placebo-controlled induction trial and 48 week, open-label extension. Lancet Gastroenterol Hepatol. 2022;7(11):1024–35. 36075249 10.1016/S2468-1253(22)00233-3

[249] SA A. Phase IIa study to evaluate the safety and efficacy of ABX464 Versus placebo in subjects with moderate to severe active ulcerative colitis who have failed or are intolerant to immunomodulators, Anti-TNFα, Vedolizumab and/or Corticosteroids. 2019, ClinicalTrials.gov

[250] SA A. Study evaluating the long-term safety and efficacy of ABX464 in active ulcerative colitis. 2022, ClinicalTrials.gov

[251] SA A. A phase 2b, open-label, efficacy and safety study of ABX464 as maintenance therapy in patients with moderate to severe ulcerative colitis. 2023, ClinicalTrials.gov

[252] SA A. Safety evaluation of ABX464 in patients with moderate to severe active Crohn's disease. 2021, ClinicalTrials.gov

[253] Hong DS, et al. Phase 1 study of MRX34, a liposomal miR-34a mimic, in patients with advanced solid tumours. Br J Cancer. 2020;122(11):1630–7. 32238921 10.1038/s41416-020-0802-1 PMC7251107

[254] Anthiya S, et al. MicroRNA-based drugs for brain tumors. Trends Cancer. 2018;4(3):222–38. 29506672 10.1016/j.trecan.2017.12.008

[255] van der Ree MH, et al. Long-term safety and efficacy of microRNA-targeted therapy in chronic hepatitis C patients. Antiviral Res. 2014;111:53–9. 25218783 10.1016/j.antiviral.2014.08.015

[256] Janssen HLA, et al. Treatment of HCV infection by targeting MicroRNA. N Engl J Med. 2013;368(18):1685–94. 23534542 10.1056/NEJMoa1209026

[257] Krishnachaitanya SS, et al. MicroRNAs in inflammatory bowel disease and its complications. Int J Mol Sci. 2022;23(15):8751. 35955886 10.3390/ijms23158751 PMC9369281

[258] Paraskevi A, et al. Circulating MicroRNA in inflammatory bowel disease. J Crohns Colitis. 2012;6(9):900–4. 22386737 10.1016/j.crohns.2012.02.006

[259] Netz U, et al. Plasma microRNA profile differentiates Crohn’s colitis from ulcerative colitis. Inflamm Bowel Dis. 2018;24(1):159–65. 10.1093/ibd/izx009 PMC5858028 29272478

[260] Chen P, et al. Circulating microRNA146b-5p is superior to C-reactive protein as a novel biomarker for monitoring inflammatory bowel disease. Aliment Pharmacol Ther. 2019;49(6):733–43. 30734320 10.1111/apt.15159

[261] Guz M, et al. Elevated miRNA inversely correlates with e-cadherin gene expression in tissue biopsies from Crohn disease patients in contrast to ulcerative colitis patients. BioMed Res Int. 2020;2020:4250329. 32775420 10.1155/2020/4250329 PMC7396102

[262] Wu W, et al. MicroRNA-206 is involved in the pathogenesis of ulcerative colitis via regulation of adenosine A3 receptor. Oncotarget. 2017;8(1):705–21. 27893428 10.18632/oncotarget.13525 PMC5352191

[263] Li J-A, et al. Downregulation of miR-214–3p may contribute to pathogenesis of ulcerative colitis via targeting STAT6. BioMed Res Int. 2017;2017:8524972. 28752100 10.1155/2017/8524972 PMC5511677

[264] Nijhuis A, et al. In Crohn’s disease fibrosis-reduced expression of the miR-29 family enhances collagen expression in intestinal fibroblasts. Clin Sci (Lond). 2014;127(5):341–50. 24641356 10.1042/CS20140048

[265] Szűcs D, et al. Increased duodenal expression of miR-146a and -155 in pediatric Crohn’s disease. World J Gastroenterol. 2016;22(26):6027–35. 27468194 10.3748/wjg.v22.i26.6027 PMC4948267

[266] Zhou H, et al. MicroRNA-223 regulates the differentiation and function of intestinal dendritic cells and macrophages by targeting C/EBPβ. Cell Reports. 2015;13(6):1149–60. 10.1016/j.celrep.2015.09.073 26526992

